# Bioengineered human brain organoids and organ-on-chip platforms for exposure-aware assessment of developmental neurotoxicity induced by general anesthetics and sedatives

**DOI:** 10.3389/fbioe.2026.1909235

**Published:** 2026-09-03

**Authors:** Jing Yue, Chenyu Pan, Chang Lu, Zhimin Song

**Affiliations:** Department of Anesthesiology, Second Hospital of Jilin University, Changchun, Jilin, China

**Keywords:** anesthetic developmental neurotoxicity, bioengineered 3D neural models, brain microphysiological systems, exposure-aware testing, human brain organoids, multimodal readouts, organ-on-chip, susceptibility-informed preclinical risk modeling

## Abstract

General anesthetic agents and sedative-hypnotic drugs are essential for fetal, neonatal, pediatric, and intensive care procedures, but their administration can overlap with vulnerable stages of human brain development. Available evidence indicates that developmental responses vary with exposure duration, repetition, developmental timing, and host susceptibility. The objective of this review is to critically evaluate bioengineered human brain organoids, brain microphysiological systems, and organ-on-chip platforms as human-relevant tools for exposure-aware assessment of anesthetic developmental neurotoxicity. We first discuss cerebral, cortical, region-specific, and patient-derived brain organoids, together with BBB- and immune-integrated chip systems, as human-relevant models for exposure-aware testing. Particular attention is given to exposure delivery, measured concentration, gas- or liquid-phase stability, microfluidic flow, matrix properties, maturation stage, recovery interval, and assay scalability. We then organize anesthetic-associated changes into platform-detectable developmental stress modules, including progenitor-zone disruption, altered lineage progression, mitochondrial and oxidative stress, ferroptotic or apoptotic injury, synaptic dysregulation, glial and neuroimmune remodeling, and network dysfunction. We further examine how divergent responses are shaped by drug class, dose, duration, brain-region identity, cellular composition, BBB or immune integration, and model architecture. Finally, we propose susceptibility-informed preclinical risk modeling based on patient-derived or isogenic organoids, multimodal readouts, biomarker anchoring, model benchmarking, and reporting standards. These platforms may support mechanism discovery, biomarker prioritization, protective-strategy screening, and translational qualification within an exposure-aware preclinical framework.

## 1 Introduction

General anesthetic agents and sedative-hypnotic drugs are indispensable in modern perioperative, procedural, and critical care medicine, yet their administration can coincide with vulnerable periods of human brain development. Clinically relevant exposure scenarios include maternal anesthesia for non-obstetric surgery during pregnancy, fetal surgery requiring direct or transplacental anesthetic administration, neonatal and pediatric surgery, diagnostic procedures, and prolonged sedation in intensive care settings. Non-obstetric surgery is estimated to be required in up to 1% of pregnancies, and open mid-gestational fetal procedures often necessitate maternal general anesthesia to provide analgesia, fetal immobilization, and uterine relaxation (Bleeser et al., 2022; Bleeser et al., 2024). After birth, preterm and term neonates increasingly undergo life-saving surgical and interventional procedures under general anesthesia, while infants and young children may require anesthesia for both operative and non-operative procedures (Houck and Vinson, 2017). These exposures are relevant to developmental neurobiology because the human brain undergoes temporally ordered processes, including neural progenitor expansion, neurogenesis, neuronal migration, synaptogenesis, gliogenesis, and early circuit refinement. In humans, rapid brain growth and synaptogenesis extend from late gestation into the first years of life, whereas fetal neurogenesis and neuronal migration are especially active during mid-gestation (Palanisamy, 2012; Bai et al., 2013). Thus, anesthetic developmental neurotoxicity is not only a pediatric anesthesia question, but also a broader problem of how pharmacological exposure is modeled during human neurodevelopment. To define the pharmacological scope of this review, we use the Anatomical Therapeutic Chemical (ATC) classification system maintained by the WHO Collaborating Centre for Drug Statistics Methodology, together with the predominant molecular targets of the drugs (WHO Collaborating Centre for Drug Statistics Methodology, 2025). The agents considered encompass general anesthetics classified under N01A and hypnotics and sedatives classified under N05C that are commonly administered in perioperative, procedural, and intensive care settings. Within N01A, the principal groups include halogenated hydrocarbons (N01AB; volatile inhalational anesthetics), represented by sevoflurane, isoflurane, and desflurane; barbiturates, plain (N01AF), represented by thiopental; and other general anesthetics (N01AX), including propofol, ketamine, etomidate, and nitrous oxide. Sedative-hypnotic agents relevant to anesthesiology include benzodiazepine derivatives (N05CD), particularly midazolam, and other hypnotics and sedatives (N05CM), including dexmedetomidine (WHO Collaborating Centre for Drug Statistics Methodology, 2025; WHO Collaborating Centre for Drug Statistics Methodology, 2026). For mechanistic comparison, these agents are additionally grouped according to their predominant, non-exclusive molecular actions, including volatile multi-target anesthetics, GABA_A_ receptor-potentiating intravenous anesthetics and benzodiazepine sedatives, N-methyl-D-aspartate receptor antagonists, and α_2_-adrenoceptor agonists (Hem et al., 2019; Weerink et al., 2017). Because the same agent may serve anesthetic or sedative roles depending on dose and clinical context, and because ATC assignment may vary according to the principal therapeutic use or route of administration, the applicable ATC category and predominant pharmacological mechanism are used throughout this review to maintain consistent terminology (WHO Collaborating Centre for Drug Statistics Methodology, 2025).

Despite 2 decades of intensive investigation, the clinical significance of anesthetic developmental neurotoxicity remains unresolved. Rodent and non-human primate studies have shown that commonly used anesthetic agents can alter immature brain development, with reported outcomes including neuronal apoptosis, impaired neurogenesis, synaptic abnormalities, mitochondrial dysfunction, and long-term behavioral or cognitive changes (Palanisamy, 2012; Bai et al., 2013). Translation of these findings to humans has been difficult. Human studies are constrained by ethical and practical limitations, heterogeneous exposure paradigms, and confounding by surgery, inflammation, underlying disease, prematurity, socioeconomic factors, and perioperative physiological instability (Useinovic and Jevtovic-Todorovic, 2023; Ing et al., 2022). The best available randomized clinical evidence from the General Anaesthesia compared to Spinal anaesthesia (GAS) trial showed that a single, short sevoflurane-based general anesthetic exposure in infancy, with a median duration of approximately 1 hour, was not associated with impaired full-scale intelligence quotient (IQ) or broad neurodevelopmental outcomes at 5 years of age (McCann et al., 2019). In contrast, observational and secondary analyses continue to raise concern that repeated or prolonged exposures may be associated with deficits in intelligence, behavior, executive function, or specific neuropsychological domains, although residual confounding cannot be excluded (Xin et al., 2025). These findings indicate that anesthetic developmental neurotoxicity should not be framed as a binary question of whether anesthetics are globally safe or toxic. More relevant questions are how drug class, dose or concentration, exposure duration, repeated exposure, developmental timing, brain-region identity, cellular lineage, physiological context, and host susceptibility shape measurable developmental responses.

These translational uncertainties create a need for experimental systems that are human-relevant, developmentally informative, and technically controllable. Conventional rodent models remain indispensable for whole-organism physiology, pharmacokinetics, maternal–fetal interaction, and behavioral assessment, but they differ from the human brain in cortical size, progenitor composition, developmental timing, regional architecture, and cellular diversity. Two-dimensional human neural cultures offer genetic accessibility and experimental control, but they lack the three-dimensional organization, spatial gradients, multicellular interactions, and tissue-level maturation needed to model complex developmental events. Human brain organoids help bridge this gap by providing three-dimensional, stem cell-derived neural tissues that can model aspects of early human brain development. Pluripotent stem cell-derived cerebral organoids can self-organize into interacting brain-region-like domains and recapitulate features such as ventricular-like progenitor zones, outer radial glia, neuronal differentiation, and neurodevelopmental disease phenotypes that are difficult to reproduce in animal models (Lancaster et al., 2013). Region-specific and bioengineered platforms, including forebrain, midbrain, and hypothalamic organoids generated in miniaturized spinning bioreactors, have improved scalability, reproducibility, and compatibility with compound testing (Qian et al., 2016; Qian et al., 2018). Further advances, such as sliced cortical organoids and fetal brain tissue-derived organoids, have extended the capacity to model cortical layer formation, sustained neurogenesis, regional identity, extracellular matrix niches, and long-term expansion (Qian et al., 2020; Hendriks et al., 2024). For anesthetic developmental neurotoxicity research, these models are valuable not simply because they are human-derived, but because their architecture and readouts allow investigators to examine how defined exposures perturb progenitor-zone organization, lineage progression, neuronal migration, synaptic maturation, glial development, mitochondrial homeostasis, and emerging network activity.

At the same time, organoids alone cannot reproduce systemic pharmacokinetics, maternal–fetal physiology, vascular perfusion, immune-endocrine signaling, cerebrospinal fluid dynamics, or behavioral outcomes. This limitation has motivated the development of brain microphysiological systems, blood–brain barrier (BBB)-integrated models, immune-integrated organoid systems, and organ-on-chip platforms. These bioengineered systems can introduce compartmentalized exposure, fluidic control, endothelial or barrier interfaces, immune-cell crosstalk, longitudinal sampling, and functional readouts that are difficult to achieve in static organoid cultures. For anesthetic and sedative studies, such design features are particularly important because the biological interpretation of an observed phenotype depends on exposure delivery, nominal *versus* measured concentration, gas or liquid stability, duration and repetition, washout interval, matrix properties, tissue size, oxygen and nutrient diffusion, and device-related adsorption. Therefore, engineering variables are not secondary technical details; they determine whether an organoid or chip experiment can be meaningfully compared with animal studies, clinical exposure scenarios, and biomarker data (Birtele et al., 2025).

**FIGURE 1:**
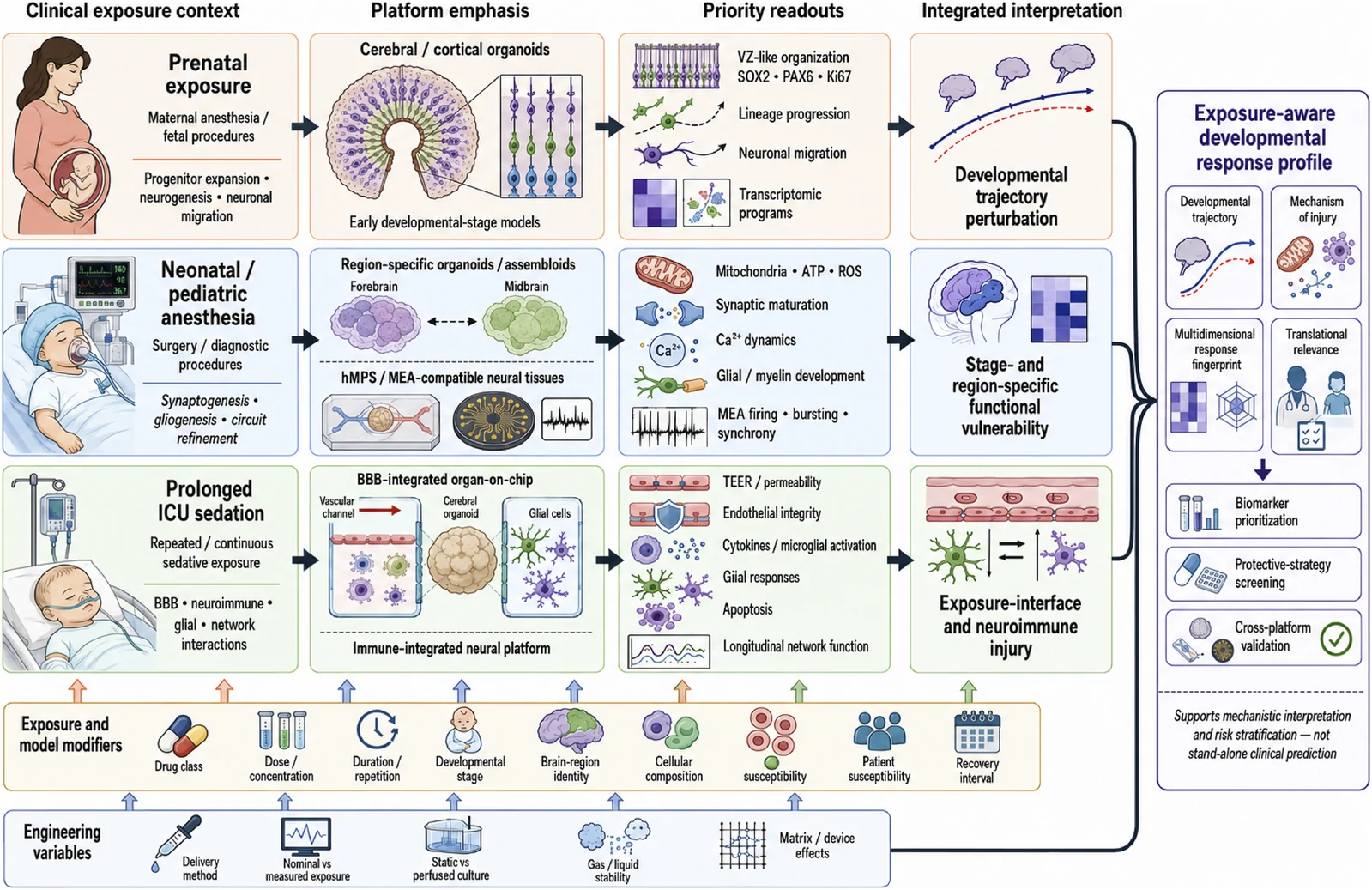
Exposure-aware platform-selection framework for assessing anesthetic developmental neurotoxicity. This schematic links clinically relevant anesthetic and sedative exposure contexts with human-relevant experimental platforms and priority readouts. Prenatal exposure is particularly suited to cerebral or cortical organoid models that interrogate progenitor-zone organization, lineage progression, neuronal migration, and developmental transcriptional programs. Neonatal and pediatric anesthesia can be examined using region-specific organoids, assembloids, and brain microphysiological systems to assess mitochondrial and metabolic stress, synaptic and glial maturation, calcium dynamics, and electrophysiological network activity. For prolonged intensive care unit (ICU) sedation, BBB-integrated or immune-integrated organ-on-chip models are particularly informative for evaluating barrier integrity, neuroimmune signaling, glial responses, apoptosis, and longitudinal network function. Across these scenarios, interpretation is modified by drug class, dose or concentration, exposure duration and repetition, developmental stage, brain-region identity, cellular composition, patient susceptibility, recovery interval, and engineering variables related to exposure delivery. These platform–readout pairings indicate experimental emphasis rather than exclusive model recommendations and converge on an exposure-aware developmental response profile for mechanistic interpretation, biomarker prioritization, protective-strategy screening, and cross-platform validation. BBB, blood–brain barrier; bMPS, brain microphysiological system; ICU, intensive care unit; MEA, microelectrode array; ROS, reactive oxygen species; TEER, transepithelial or transendothelial electrical resistance.

Unlike previous reviews that primarily summarize anesthetic mechanisms or clinical outcomes, this review treats anesthetic developmental neurotoxicity as an application and validation domain for bioengineered human brain organoids, brain microphysiological systems, and organ-on-chip platforms. The objective of this review is to critically evaluate how these human-relevant 3D neural platforms can be designed, applied, benchmarked, and qualified for exposure-aware assessment of developmental neurotoxicity induced by general anesthetics and sedatives. Specifically, we aim to link clinically relevant exposure conditions with appropriate model selection and multimodal readouts, distinguish shared from agent-, stage-, and region-specific developmental injury mechanisms, and assess how these platforms may support susceptibility-informed preclinical risk modeling, biomarker prioritization, and protective-strategy screening. Accordingly, the review is organized around the interaction between exposure conditions, developmental context, and model capability, rather than around individual anesthetic agents or isolated injury pathways alone. We first summarize cerebral, cortical, region-specific, microphysiological, BBB-integrated, and immune-integrated platforms, with attention to exposure modeling and multimodal readout design. We then reorganize anesthetic-associated changes into platform-detectable developmental stress modules, including progenitor-zone disruption, altered lineage progression, mitochondrial and oxidative stress, programmed cell death, synaptic dysregulation, glial and neuroimmune remodeling, and network dysfunction. We further examine how biological and engineering variables, including drug class, dose or concentration, exposure paradigm, developmental stage, brain-region identity, cellular composition, model architecture, and recovery interval, shape divergent responses across experimental systems. Finally, we discuss how patient-derived or isogenic organoids, multi-omics profiling, electrophysiological assessment, biomarker anchoring, cross-platform benchmarking, and standardized reporting may support susceptibility-informed preclinical risk modeling. Together, these applications position human 3D neural models within a translational workflow spanning mechanism discovery, candidate biomarker prioritization, protective-strategy screening, and model qualification. This bioengineered workflow, from clinical exposure context to human-relevant platforms, multimodal readouts, and exposure-aware interpretation, is summarized in Figure 1.

## 2 Literature search and review methodology

This narrative review was supported by a structured literature search. PubMed was searched without a predefined restriction on publication date, and the literature search was updated through July 2026. Search terms were used alone or in combination and covered three major domains: (1) general anesthetics and sedatives, including “sevoflurane,” “isoflurane,” “desflurane,” “propofol,” “ketamine,” “thiopental,” “midazolam,” and “dexmedetomidine”; (2) developmental neurotoxicity and related outcomes, including “developmental neurotoxicity,” “neurodevelopment,” “neuroapoptosis,” “mitochondrial dysfunction,” “oxidative stress,” “neuroinflammation,” “synaptic dysfunction,” and “cognitive impairment”; and (3) human-relevant experimental platforms, including “brain organoid,” “cerebral organoid,” “neural organoid,” “brain microphysiological system,” “organ-on-chip,” “brain-on-chip,” and “blood-brain barrier.” The reference lists of relevant primary studies and review articles were also examined to identify additional publications of direct relevance to the topics covered.

Studies were selected on the basis of their relevance to the pharmacology, developmental toxicodynamics, molecular and cellular mechanisms, and neurodevelopmental outcomes associated with exposure to general anesthetics or sedatives. Particular attention was given to studies using human brain organoids, region-specific organoids, brain microphysiological systems, and organ-on-chip platforms. Because human 3D evidence remains limited for several anesthetic agents and mechanistic domains, relevant two-dimensional neural models, animal studies, clinical studies, and authoritative reviews were also included when they provided mechanistic, pharmacological, toxicodynamic, or translational information necessary for interpretation. Recent studies were prioritized where available, while earlier studies were retained when they provided foundational or otherwise unique evidence.

The initial literature identification and relevance assessment were performed by the primary reviewer. During manuscript preparation and revision, key studies included in the review and the corresponding mechanistic and translational interpretations were independently cross-checked by a second author. Discrepancies regarding study relevance, interpretation, or the strength of evidence were resolved through discussion and, where necessary, consultation with the corresponding author. Particular attention was paid to consistency between the cited evidence and statements presented in the main text, figures, and tables. Because the present article is a narrative review integrating heterogeneous evidence from pharmacological, bioengineering, *in vitro*, animal, and clinical studies, no formal meta-analysis or quantitative risk-of-bias assessment was performed.

## 3 Bioengineered human brain organoid and brain microphysiological platforms for anesthetic exposure assessment

### 3.1 Cerebral, cortical, and region-specific brain organoids

Across bioengineered human neural platforms, the central aim is not to determine whether an anesthetic is universally safe or toxic, but to define how developing human neural tissues respond under controlled exposure conditions. Human brain organoids are useful developmental testbeds because they preserve aspects of early human neurogenesis that are difficult to reproduce in conventional two-dimensional cultures or animal models. Pluripotent stem cell-derived cerebral organoids can self-organize into three-dimensional neuroepithelial tissues containing ventricular zone-like regions, radial glial progenitors, intermediate progenitors, and differentiating neurons, thereby recapitulating progenitor expansion, neuronal differentiation, migration-related architecture, and early cortical patterning (Lancaster et al., 2013). This spatial organization is directly relevant to anesthetic exposure assessment, because many potential injury signals may appear as changes in progenitor-zone integrity, lineage timing, neuronal migration, synaptic maturation, or early network activity rather than as overt neuronal loss alone.

Unguided cerebral organoids are particularly suitable for broad developmental screening because they generate heterogeneous neural tissues with cortical-like domains and multiple neural cell populations. Early characterization studies showed that induced pluripotent stem cell (iPSC)-derived cerebral organoids progressively express neuronal, astrocytic, vascular-like, channel-related, and synaptic genes, and can exhibit action potentials, multiple channel activities, and functional responses to propofol (Logan et al., 2020). These features support their use not only as structural models of early human brain development, but also as functional platforms for detecting anesthetic-sensitive changes in excitability and channel-associated maturation. Cortical organoids refine this approach by focusing on dorsal telencephalic development, ventricular and subventricular zone organization, radial glial dynamics, and early cortical differentiation. In human embryonic stem cell (hESC)-derived cerebral organoids exposed to sevoflurane during a first-trimester-equivalent window, sevoflurane transiently reduced SOX2^+^/N-cadherin^+^ ventricular zone-like structures and increased early neuronal markers such as TUJ1 and MAP2, with partial recovery at later stages (Lee et al., 2022). Related work further suggests that sevoflurane can perturb radial glial interkinetic nuclear migration and Notch-associated developmental regulation (Jiang et al., 2021). Therefore, cortical organoids are especially useful for evaluating progenitor-zone organization, radial glial behavior, neurogenic timing, and developmental-trajectory shifts.

Region-specific organoids and spheroids add another layer of platform resolution by allowing anesthetic responses to be mapped onto defined brain-region identities and lineage programs. Dorsal forebrain or cortical spheroids primarily model excitatory cortical neuron development, whereas ventral forebrain or subpallial spheroids generate interneuron-like lineages and can be assembled with dorsal spheroids to study migration and excitatory–inhibitory circuit formation (Birey et al., 2017). This regionalization is important because anesthetic effects may differ across brain regions, neuronal subtypes, and maturation states. Recent propofol studies illustrate this principle. Human dorsal and ventral forebrain organoids showed region-dependent differences in neuronal differentiation, maturation, metabolic adaptation, and electrophysiological activity after exposure (She et al., 2026), while intermittent propofol exposure in human brain organoids increased neuronal activity and upregulated neurodevelopmental and synaptic genes after early, but not later, exposure (Wang et al., 2026). These findings indicate that forebrain organoid and assembloid systems are well suited for detecting regional identity, excitatory–inhibitory balance, lineage maturation, and early circuit-level endpoints.

Midbrain-like organoids further extend this strategy to dopaminergic lineage development. In human iPSC-derived midbrain organoids, prolonged sevoflurane exposure promoted premature dopaminergic differentiation, whereas shorter exposure produced weaker effects (Shang et al., 2022). This example shows how region-specific platforms can reveal lineage-selective vulnerability that may be diluted or missed in more heterogeneous cerebral organoids. Cerebral organoids have also been used to model ketamine-related exposure, with chronic high-concentration (2R,6R)-hydroxynorketamine suppressing neural precursor expansion, increasing progenitor apoptosis, and altering radial glial division orientation (Du et al., 2023). In addition, organoid-derived human neurons can be combined with *in vivo* environments: human neuronal chimeric mice revealed that sevoflurane impaired human neuronal migration through DVL-1/Ca^2+^ signaling, and that chemogenetic activation, DVL-1 overexpression, or repetitive transcranial magnetic stimulation partially rescued migration and social phenotypes (Zhao et al., 2025). Together, these studies show that cerebral, cortical, and region-specific organoids are most informative when anesthetic exposure is interpreted in relation to drug class, dose, duration, developmental stage, regional identity, and cellular lineage (Xiao et al., 2024; Chen et al., 2026). In this platform-oriented framework, organoids should be selected according to the developmental endpoint being tested, such as progenitor-zone integrity, regional patterning, lineage maturation, neuronal migration, synaptic development, or emerging network activity.

### 3.2 Brain microphysiological systems and BBB- and immune-integrated organ-on-chip designs

Although cerebral and region-specific organoids capture important aspects of intrinsic neural development, static organoid cultures do not fully reproduce the exposure routes and tissue interfaces encountered in clinical anesthesia or intensive care sedation. *In vivo*, anesthetics and sedatives reach the developing brain through vascular transport, tissue diffusion, blood–brain barrier (BBB) permeability, systemic metabolism, inflammatory signaling, and repeated or prolonged dosing. Brain microphysiological systems (bMPSs) and organ-on-chip platforms extend organoid models by introducing standardized three-dimensional neural microtissues, compartmentalized culture formats, microfluidic flow, barrier interfaces, immune-cell crosstalk, and longitudinal sampling. These design features are central to anesthetic exposure assessment because they allow investigators to separate direct neural effects from endothelial, glial, immune, and barrier-associated responses.

Human iPSC-derived bMPS platforms are typically designed as reproducible three-dimensional neural microtissues containing neural progenitors, neurons, astrocytes, and oligodendrocyte lineage cells. Compared with larger and more heterogeneous unguided organoids, these systems can improve assay scalability and reduce some sources of batch-to-batch variability while preserving key developmental processes, including proliferation, apoptosis, differentiation, migration, neurite outgrowth, synaptogenesis, myelination, and early network formation. In a sevoflurane-bMPS study, human iPSC-derived neural progenitors were differentiated into brain-like spheroids, exposed to 2.4% sevoflurane for 4 h at week 8, and analyzed 4 weeks later. Sevoflurane reduced bMPS size, increased cleaved caspase-3-positive cells, decreased neural differentiation markers, reduced mature neuron/neural progenitor and mature oligodendrocyte/oligodendrocyte precursor cell ratios, impaired myelination, disrupted astrocyte development and excitatory synaptogenesis, and increased mTOR pathway activity (Li et al., 2026). This delayed analysis illustrates a key advantage of bMPS design: it can detect persistent developmental consequences after a defined exposure and recovery interval, rather than limiting assessment to acute cytotoxicity.

Functional microphysiological systems further expand this platform logic. Microelectrode array (MEA)-compatible systems, such as the BrainSphere high-density MEA assay, use human iPSC-derived three-dimensional neural microtissues containing neurons, astrocytes, and oligodendrocytes, and record spontaneous firing and network bursting from 7-week-old tissues during multi-concentration, multi-day exposure (Carstens et al., 2025). Although this platform was developed for environmental neurotoxicity screening, it is highly relevant to anesthetic studies because many anesthetic and sedative agents alter excitability, receptor signaling, synchronization, and network maturation. MEA-compatible bMPSs therefore provide a functional toxicology layer that complements structural, molecular, and metabolic endpoints. In anesthetic developmental neurotoxicity research, such systems are particularly useful for distinguishing transient suppression of activity from delayed disruption of network formation after washout or recovery.

BBB modeling adds another clinically important engineering component. The BBB restricts the entry of cells, pathogens, chemicals, and toxins into the brain through tight junctions, efflux transporters, endothelial specialization, and interactions with supporting neural and glial cells. Human iPSC-derived BBB models are increasingly used to assess central nervous system penetration and barrier disruption, but they require careful validation because strong barrier function does not always indicate mature brain endothelial identity. Transcriptomic benchmarking is therefore essential when BBB models are used to interpret drug permeability, endothelial injury, or barrier-mediated neurotoxicity (Wellens et al., 2022). For anesthetic exposure assessment, BBB-integrated systems are valuable because they can model vascular-side delivery, barrier permeability, transendothelial electrical resistance (TEER), and endothelial-neural communication, all of which are absent from standard static organoid cultures.

Organ-on-chip models integrate these elements within compartmentalized devices. A vascular chamber lined with brain endothelial cells can be separated from a neural tissue chamber containing organoids, glia, immune cells, and extracellular matrix, allowing drugs to be delivered through a vascular-like route rather than being added directly to the neural compartment. The ICU patient-on-a-chip platform exemplifies this design. In this model, human cerebral microvascular endothelial cells formed a BBB-like membrane interface, while iPSC-derived mast cells and cerebral organoids were embedded in a three-dimensional matrix to emulate prolonged ICU sedation. Propofol (200 μM) or midazolam (25 μM) was administered through the co-culture medium and repeated on day 2, with responses analyzed at the end of day 4. These exposures produced distinct changes in BBB permeability, transendothelial electrical resistance, endothelial integrity, mast-cell activation, microglial and macroglial markers, cytokine expression, glutamate-associated toxicity, receptor-related genes, and apoptotic zones in cerebral organoids (Saglam-Metiner et al., 2024a). These findings show that sedative-associated developmental neurotoxicity may involve endothelial–immune–glial–neuronal interactions rather than uniform direct neuronal injury.

Beyond its biological outputs, this platform also illustrates why microfluidic operating conditions should be reported quantitatively. In the ICU patient-on-a-chip system, oscillatory motion at 1 rpm generated a flow rate of approximately 18 μL/s in the vascular compartment. Computational fluid-dynamic analysis estimated a maximum velocity of 7.73 × 10^−4^ m/s and a maximum wall shear stress of 6.88 × 10^−4^ Pa under this condition (Saglam-Metiner et al., 2024a). Increasing the rocking speed to 5 and 10 rpm increased the maximum shear stress to 2.97 × 10^−3^ and approximately 7 × 10^−3^ Pa, respectively. When the three-dimensional matrix surrounding the organoids was incorporated into the simulation, the estimated maximum shear stress was reduced to approximately 0.06 × 10^−3^ Pa (Saglam-Metiner et al., 2024a). These differences illustrate that flow-driving method, channel geometry, matrix architecture, and operating speed can substantially alter the mechanical environment experienced by endothelial and neural compartments. Future bMPS and organ-on-chip studies should therefore report flow rate or velocity, channel dimensions, flow regime, and calculated or measured shear stress rather than describing the system generically as “perfused.”

Device material represents a second source of exposure uncertainty. The ICU patient-on-a-chip platform is fabricated primarily from polydimethylsiloxane (PDMS), with a polyethylene terephthalate membrane separating the vascular and neural compartments (Saglam-Metiner et al., 2024a). PDMS is optically transparent, flexible, and gas permeable, but it can sorb hydrophobic small molecules onto its surface and into its bulk, thereby changing the freely available drug concentration and producing delayed release during washout. A recent quantitative comparison of PDMS and cyclic olefin copolymer (COC) microfluidic devices demonstrated that sorption was strongly dependent on compound physicochemical properties. After 24 h of exposure to an initial concentration of 100 μM, the highly lipophilic compound imipramine (logP = 4.80) decreased to 0.0384 μM in PDMS devices, compared with 31.5 μM in COC devices (Grindulis et al., 2025). These values should not be extrapolated directly to propofol, midazolam, or other anesthetics, but they demonstrate that nominal inlet concentration may differ markedly from the concentration actually available within a microfluidic system. For lipophilic anesthetic and sedative agents, future studies should therefore quantify drug recovery at the inlet and outlet and, where feasible, in culture medium and neural tissue, while incorporating material-only sorption controls.

Mass transport and oxygenation represent a third engineering constraint. Conventional avascular cerebral organoids develop diffusion-limited oxygen and nutrient transport as tissue size increases; oxygen, nutrient, and waste exchange from the culture medium becomes limited at approximately 400 μm from the organoid surface (Castiglione et al., 2022). This can generate spatial oxygen gradients and hypoxic or necrotic cores. Such effects are particularly relevant to anesthetic developmental neurotoxicity because hypoxia can independently alter mitochondrial function, reactive oxygen species production, progenitor behavior, cell death, and neuronal maturation, potentially confounding endpoints attributed to anesthetic exposure. Perfusion, tissue miniaturization, slicing, air–liquid-interface culture, and vascularized designs can improve mass transport, but they also introduce different mechanical and exposure environments. Organoid and chip studies should therefore report tissue dimensions, oxygenation conditions, perfusion parameters, and, where technically feasible, spatial oxygen measurements together with drug-exposure data.

Despite these advantages, bMPS and organ-on-chip systems remain simplified models. They cannot fully reproduce placental transfer, hepatic metabolism, endocrine signaling, cerebrospinal fluid dynamics, organism-level compensation, or long-term behavioral outcomes. Importantly, engineering parameters should be treated as experimental variables rather than as secondary methodological details. Differences in flow regime, device material, tissue geometry, matrix composition, barrier maturity, oxygenation, sampling schedule, and recovery interval can alter both effective anesthetic exposure and baseline cellular physiology. Cross-platform comparison therefore requires these parameters to be reported together with nominal and, where feasible, measured drug concentrations and biological readouts. BBB- and immune-integrated chip systems should consequently be viewed as exposure-aware platforms for mechanistic dissection, exposure modeling, and cross-platform benchmarking.

### 3.3 Multimodal readouts for developmental neurotoxicity

The usefulness of organoid-based platforms ultimately depends on how readouts are selected and timed. Because anesthetic developmental neurotoxicity may appear as altered maturation, network imbalance, mitochondrial stress, or inflammatory activation rather than gross tissue loss, no single endpoint is sufficient. Morphological readouts such as organoid size, ventricular zone integrity, neural rosette organization, radial arrangement, and cell distribution provide an initial assessment of tissue architecture. Immunofluorescence can then quantify progenitors (SOX2, PAX6, NESTIN, Ki67), intermediate progenitors (TBR2/EOMES), neurons (TUJ1, DCX, MAP2, NEUN), cortical identities (TBR1, CTIP2, SATB2), astrocytes (GFAP, S100B), oligodendrocytes and myelin (OLIG2, O4, MBP), apoptosis (cleaved caspase-3, TUNEL), and autophagy (LC3, p62). Practical organoid neurotoxicity protocols emphasize that neurogenesis, apoptosis, calcium imaging, MEA recording, and immunostaining can be combined into an accessible assay battery (Parmentier et al., 2023). For anesthetic studies, these markers should be interpreted as platform readouts of progenitor-zone integrity, lineage progression, glial maturation, and developmental trajectory, rather than as isolated injury signals.

Targeted qPCR and Western blotting validate specific pathways, whereas bulk RNA-seq, microarray analysis, lncRNA profiling, and single-cell RNA-seq define broader molecular programs and cell-type-specific responses. Baseline transcriptomic maps of organoid maturation are essential because the same gene change may reflect toxicity, delayed maturation, or accelerated maturation depending on developmental context (Logan et al., 2020). In propofol-exposed organoids, transcriptomic and electrophysiological readouts suggested early-stage acceleration of maturation-associated genes and neural activity (Wang et al., 2026), while region-specific propofol studies linked transcriptional adaptation to dorsal–ventral functional differences (She et al., 2026). Integrated organoid–patient studies add translational anchoring: propofol exposure dysregulated mRNAs and lncRNAs related to synaptic function, mitochondrial activity, and inflammation in organoids, reduced ATP production and CKMT1B expression, and showed overlapping RNA signatures in serum from children exposed to prolonged anesthesia (Pant et al., 2026). Broader toxicology studies also show how cerebral organoids support cell-type-resolved or network-level interpretation, including PFAS-induced neuron-associated Alzheimer-like pathology and lipidomic disruption (Lu et al., 2024) and tetrodotoxin-induced changes in voltage-gated channel and synaptic homeostasis networks (Liu et al., 2023).

Functional readouts are particularly important for anesthetic and sedative assessment because many agents directly alter excitability, receptor signaling, and network synchrony. Calcium imaging detects single-cell or local population activity, whereas MEA recording measures firing rate, bursting, synchrony, and network organization over time. High-density MEA platforms improve spatial coverage and can detect functional perturbations before obvious morphological injury (Carstens et al., 2025). Patch-clamp electrophysiology provides cell-level measures of membrane properties, action potentials, synaptic currents, and drug responses, complementing network-level assays (Logan et al., 2020). Mitochondrial and metabolic assays, including ATP production, mitochondrial morphology, membrane potential, reactive oxygen species, lactate release, and respiratory protein expression, capture another major injury axis. Finally, serum biomarkers such as neuronal injury markers, glial markers, neurotrophic factors, and circulating RNAs may help link organoid phenotypes to clinical exposure groups, although larger validation cohorts are needed (Pant et al., 2026). Overall, the strongest studies will combine structural, molecular, electrophysiological, metabolic, and biomarker-oriented readouts across defined exposure and recovery windows. This multimodal framework supports comparison across anesthetic classes, distinction between transient adaptation and persistent injury, and prioritization of mechanisms for validation in animal models and perioperative cohorts. Because nominal exposure does not necessarily correspond to the concentration experienced by neural tissue, cross-study comparisons require explicit consideration of exposure engineering and internal dosimetry. This distinction is particularly important for volatile anesthetics, for which the administered gas-phase concentration cannot be assumed to equal the dissolved-medium or tissue concentration, and for lipophilic intravenous agents, for which tissue accumulation may change substantially over the course of exposure (Lee et al., 2022; Wang et al., 2026). Accordingly, Table 1 distinguishes nominal exposure concentration, exposure duration and recovery interval, administration or delivery format, and measured or estimated internal exposure where such information was reported. When internal exposure was not directly quantified, this is explicitly indicated rather than inferred from the nominal concentration. Representative organoid-based and microphysiological studies are summarized in Table 1.

**TABLE 1:** Representative human brain organoid-based and microphysiological studies of anesthetic developmental neurotoxicity with exposure-engineering parameters.

Platform/model	Exposure paradigm	Administration/delivery	Internal exposure/dosimetry	Developmental context	Major readouts/findings	Refs
hESC-derived cerebral organoids	Sevoflurane, 6, 12, or 30 vol%; 6 h on D11–12; later developmental follow-up	Gas-phase exposure of organoid cultures	Not directly measured. Values represent nominal gas-phase concentrations; 6 vol% was selected by the authors using water/gas versus blood/gas partitioning as a dosing rationale	First-trimester-equivalent cerebral development	VZ-like structures, SOX2/N-cadherin, TUJ1, MAP2, cell death; transient VZ-like reduction and increased early neuronal differentiation followed by partial recovery	(Lee et al., 2022)
hESC-derived cerebral organoids with fetal-cortex validation	Sevoflurane, 4.1 vol%; 6 h at approximately D30	Gas-phase exposure of organoid cultures	Not directly measured; nominal gas-phase concentration only	Radial glial progenitor development	INM and Notch/Jag1/NICD signaling; temporary impairment of radial glial INM	(Jiang et al., 2021)
Dorsal and ventral forebrain organoids	Propofol, 20 μM; single 6-h exposure on D11; longitudinal analysis through D80	Direct organoid culture exposure; non-microfluidic	Not directly measured. The nominal 20 μM concentration was selected to approximate clinically relevant unbound plasma propofol concentrations	Early dorsal and ventral forebrain development	Region- and time-dependent effects on growth, differentiation, metabolism, neuronal identity, and electrophysiological maturation	(She et al., 2026)
hESC-derived human brain organoids	Propofol, 50 μM; 72 h at D47–50 or D77–80; followed by 10-day drug-free recovery	Direct addition to culture medium in orbital-shaker organoid culture	Directly measured by GC–MS: 172.9 ± 43 ng/mg organoid at 1 h and 6.1 ± 1.2 ng/mg at 72 h	Early versus later developmental exposure windows	Growth, lactate release, transcriptomics, synaptic programs, and HD-MEA activity; early exposure produced stronger developmental and functional alterations	(Wang et al., 2026)
hiPSC-derived midbrain organoids	Sevoflurane, 2 vol%; 2 or 6 h during early differentiation	Gas-phase exposure of organoid cultures	Not directly measured; nominal gas-phase concentration only	Developing dopaminergic midbrain	Progenitor and dopaminergic markers, proliferation and transcriptomics; 6-h exposure promoted premature dopaminergic differentiation, whereas shorter exposure produced weaker effects	(Shang et al., 2022)
hESC-derived cerebral/forebrain organoids	(2R,6R)-HNK, 20 or 100 μM; 3- or 12-day exposure depending on developmental window	Direct culture-medium exposure; non-microfluidic	Directly measured by LC–MS: mean organoid contents of 27.4 and 60.5 ng/g after 20 and 100 μM exposure, respectively	Early cortical progenitors and radial glia	NPC proliferation, cell death, cortical organization, and radial glial division orientation; effects were strongest at the higher concentration	(Du et al., 2023)
Human iPSC-derived bMPS	Sevoflurane, 2.4 vol%; 4 h at week 8; 4-week post-exposure recovery	Airtight chamber connected to an anesthetic vaporizer; 15-min equilibration with 2.4% sevoflurane in 5% CO2/95% air, followed by 4-h exposure under gyratory shaking	Not directly measured; nominal gas-phase concentration only	Multilineage neural microtissues at week 8	Persistent apoptosis, altered neural and oligodendrocyte differentiation, impaired myelination, astrocyte and synaptic abnormalities, and increased mTOR signaling	(Li et al., 2026)
BBB–mast cell–cerebral organoid chip	Propofol, 200 μM, or midazolam, 25 μM; administered on days 1 and 2; analysis at end of day 4	Drug administration through flowing co-culture medium in the vascularization layer of a microfluidic BBB–immune–organoid device; organoid-only well-plate comparator also evaluated	Not directly measured; nominal co-culture-medium concentrations only	ICU sedation-like BBB–immune–neural interface	BBB permeability, TEER, endothelial integrity, mast-cell activation, glial responses, cytokines, glutamate-associated toxicity, receptor genes, and apoptosis	(Saglam-Metiner et al., 2024a)
Day-60 iPSC-derived cerebral organoids + pediatric serum	Propofol: single 10 μg/mL (56 μM) for 1, 2, 3, or 6 h; or repeated 5 or 10 μg/mL (28 or 56 μM) for 1 or 2 h/day for 3 consecutive days; mechanistic profiling used 10 μg/mL for 6 h	Direct culture exposure of cerebral organoids; non-microfluidic	Not directly measured in organoids. Dosing was contextualized against reported pediatric blood concentrations of 1–10 μg/mL and estimated human brain tissue concentrations of 4–20 μg/mL (22.4–112 μM)	Day-60 cerebral organoids with clinical serum anchoring	Apoptosis/autophagy, mRNA/lncRNA profiles, mitochondrial ultrastructure, ATP, CKMT1B, PSD95/c-Fos, and overlapping circulating RNA signatures	(Pant et al., 2026)

Exposure values are reported as stated in the original studies. For volatile anesthetics, percentages refer to nominal gas-phase concentrations unless otherwise specified and should not be interpreted as equivalent dissolved-medium or tissue concentrations. “Not directly measured” indicates that the original study did not quantify drug concentration within the neural tissue. Where gas-phase doses were selected using partition-coefficient considerations, or nominal liquid-phase doses were contextualized using clinical plasma or brain-tissue estimates, these values are reported only as dosing rationales and should not be interpreted as measured organoid exposure. BBB, blood–brain barrier; bMPS, brain microphysiological system; GC–MS, gas chromatography–mass spectrometry; HD-MEA, high-density microelectrode array; hESC, human embryonic stem cell; hiPSC, human induced pluripotent stem cell; HNK, hydroxynorketamine; INM, interkinetic nuclear migration; NPC, neural progenitor cell; TEER, transepithelial or transendothelial electrical resistance; VZ, ventricular zone.

### 3.4 Validation of structural, cellular, electrophysiological, and circuit fidelity

Human brain organoids should not be interpreted as miniature replicas of the human brain. Their fidelity is domain- and maturation-dependent: current models reproduce selected features of fetal and, after prolonged culture, early postnatal brain development with considerably greater fidelity than they reproduce mature whole-brain organization. Structurally, cerebral and cortical organoids can generate neuroepithelial and ventricular zone-like regions, radial glial progenitors, intermediate progenitors, neuronal layers, and region-associated developmental identities (Lancaster et al., 2013; Qian et al., 2020). Direct comparison with human fetal tissue provides stronger validation at the molecular level. Single-cell transcriptomic analysis showed that cerebral organoids reproduce gene-expression programs associated with progenitor proliferation and neuronal differentiation in the human fetal neocortex (Camp et al., 2015). In a separate study of 166,242 cells from 21 dorsal forebrain organoids, 95% of individual organoids generated a highly similar repertoire of cortical cell types, and their developmental trajectories were transcriptionally similar to those of the human fetal cortex (Velasco et al., 2019). These findings support substantial fidelity for selected early cortical cell identities and developmental programs, but not for the complete cellular composition or macroscopic anatomy of the human brain. Standard organoids may incompletely represent vascular, immune, mesoderm-derived, and late-maturing glial populations, and their spatial organization remains simpler and more variable than that of the intact developing brain (Birtele et al., 2025; Wang et al., 2023).

Electrophysiological fidelity is also partial but experimentally demonstrable. Cerebral organoids can develop functional ion channels, action potentials, synaptic activity, and pharmacological responsiveness, as already demonstrated in the structural and electrophysiological characterization study cited above (Logan et al., 2020). More mature organoids have been shown to form dendritic spines and electrically active neuronal networks that respond to sensory-like photostimulation (Quadrato et al., 2017). Long-term cortical organoids can also generate increasingly complex spontaneous oscillatory activity, including network-synchronous events and phase-amplitude coupling. Trujillo et al. reported that developmental changes in these network dynamics shared quantitative features with electroencephalographic patterns observed in preterm human neonates (Trujillo et al., 2019). Importantly, such similarities indicate developmental correspondence in selected electrophysiological features and should not be interpreted as equivalence between organoid activity and whole-brain human electroencephalography. Prolonged culture can further extend maturation: cortical organoids maintained for approximately 250–300 days displayed transcriptomic, epigenetic, RNA-editing, and N-methyl-D-aspartate receptor transitions associated with early postnatal development (Gordon et al., 2021).

Functional connectivity can be modeled at local and inter-regional levels, but remains far from whole-brain connectivity. Fusion of dorsal and ventral forebrain spheroids reproduces saltatory interneuron migration from ventral to dorsal forebrain domains, after which migrated interneurons functionally integrate with glutamatergic neurons to form local microcircuits (Birey et al., 2017). More advanced air–liquid-interface cerebral organoids generate long-range axonal tracts showing projection, growth-cone turning, and decussation; these tracts can produce functional output when connected to extracerebral target tissues (Giandomenico et al., 2019). Transplantation provides an additional validation layer: human brain organoids transplanted into mouse brain undergo further neuronal and glial maturation, become vascularized, extend axons into host regions, exhibit functional neuronal activity, and show evidence of graft-to-host synaptic connectivity (Mansour et al., 2018). Nevertheless, standard *in vitro* organoids do not reproduce the full human connectome, mature thalamocortical and corticocortical organization, sensory inputs, neuromodulatory systems, or the distributed network architecture required for cognition and behavior. Thus, organoid connectivity is best interpreted as local or modular circuit competence rather than whole-brain functional equivalence.

Taken together, current validation studies indicate that brain organoids reproduce selected developmental features of the human brain rather than the brain as a complete organ. Their strongest validity domains are early developmental gene programs, progenitor organization, neuronal lineage specification, and increasingly mature local electrophysiological and synaptic functions. Fidelity decreases as the question moves toward mature cellular diversity, vascular and immune integration, long-range systems-level connectivity, and organism-level function. For developmental anesthetic neurotoxicity, this distinction is particularly important: organoids are suitable for testing whether defined exposures alter human progenitor dynamics, neuronal and glial differentiation, synaptic maturation, excitability, and local network organization, whereas cognition, behavior, and whole-brain functional connectivity require complementary whole-organism and clinical evidence. Accordingly, organoid phenotypes should be benchmarked against human fetal or postnatal reference datasets and integrated with electrophysiological, animal, and clinical evidence rather than used as stand-alone surrogates of the intact human brain.

## 4 Platform-detectable convergent endpoints and developmental stress modules

### 4.1 Progenitor-zone organization, lineage allocation, and developmental trajectory endpoints

In organoid and brain microphysiological platforms, altered neural progenitor behavior should be interpreted not only as a mechanism of anesthetic developmental neurotoxicity, but also as one of the most accessible developmental trajectory endpoints. During early human corticogenesis, neural progenitor cells (NPCs), radial glial cells, and intermediate progenitors coordinate self-renewal, neurogenic commitment, apical mitosis, interkinetic nuclear migration (INM), and neuronal migration. Perturbation of these processes can change the timing and output of neurogenesis, leading to altered regional cell composition, laminar organization, and subsequent circuit assembly. This perspective moves the field beyond an apoptosis-centered model and makes progenitor-zone architecture, lineage allocation, and developmental timing key readouts for human 3D neural platforms. Human stem cell-based systems were proposed as useful tools for interrogating anesthetic effects on human neural proliferation and neurogenesis, because they allow controlled assessment of lineage-specific vulnerability during periods of rapid neural growth (Bai et al., 2013).

Evidence from different anesthetic classes supports this trajectory-based endpoint framework. In fetal rat neural stem/progenitor cells, clinically relevant ketamine exposure did not induce apoptosis or necrosis, but inhibited proliferation, reduced BrdU- and Ki67-positive cells, and increased TUJ1-positive neuronal differentiation (Dong et al., 2012). Thus, altered neurogenesis can occur without overt cell death, suggesting that anesthetic exposure may shift progenitor fate decisions rather than simply eliminate neural cells. Similar principles are detectable in organoid models of volatile anesthetic exposure. In hESC-derived cerebral organoids exposed to sevoflurane during a first-trimester-equivalent window, intermediate exposure did not significantly change overall organoid size or induce prominent cell death, but transiently reduced SOX2^+^/N-cadherin^+^ ventricular zone-like structures and temporarily increased early neuronal markers, including TUJ1 and MAP2 (Lee et al., 2022). Although these markers partially normalized at later stages, the findings indicate that cerebral organoids can capture transient acceleration of neuronal differentiation and destabilization of progenitor-zone organization during a sensitive developmental window.

Radial glial biology represents a particularly important platform-detectable vulnerability node. Radial glial progenitors are stem-like cells, scaffolds for neuronal migration, and organizers of cortical tissue geometry. Maternal sevoflurane exposure induced temporary defects in INM of radial glial progenitors in the fetal cerebral cortex and hESC-derived brain organoids through a Notch-dependent mechanism; activation of Jag1/NICD signaling mitigated this defect (Jiang et al., 2021). Because INM is closely coupled to apical mitosis and the balance between proliferative and neurogenic divisions, its disruption can be evaluated as a readout of progenitor pool maintenance and cortical output. Region-specific organoid studies suggest that this developmental endpoint is not limited to cortical-like tissues. In hiPSC-derived midbrain organoids, prolonged sevoflurane exposure reduced progenitor features and promoted premature dopaminergic differentiation, reflected by enhanced MAP2 and tyrosine hydroxylase expression, whereas shorter exposure produced weaker effects (Shang et al., 2022). These findings show how region-specific organoids can detect premature lineage maturation in defined neural lineages.

Intravenous anesthetics and related compounds also converge on progenitor and differentiation programs, although the direction and regional expression of the response may differ. Propofol has been associated with altered proliferation, migration, neurogenesis, and maturation in preclinical systems, and recent forebrain organoid work showed that propofol produced region-specific effects in human dorsal and ventral forebrain organoids, including differences in neuronal differentiation, metabolic adaptation, and electrophysiological maturation (She et al., 2026). Another human brain organoid study found that intermittent propofol exposure altered neurodevelopmental and synaptic gene programs in an exposure-stage-dependent manner, with early exposure producing stronger effects than later exposure (Wang et al., 2026). Ketamine-related exposure is also relevant: chronic high-concentration exposure to (2R,6R)-hydroxynorketamine suppressed neural precursor expansion, increased progenitor apoptosis, and altered radial glial division orientation in cerebral organoids (Du et al., 2023). These studies indicate that different anesthetic agents may converge on a shared developmental axis involving progenitor maintenance, division mode, and differentiation timing, even when their molecular targets and phenotypic profiles are not identical.

The consequences of altered progenitor behavior extend downstream to neuronal migration and cortical patterning, which are also measurable in human-relevant platform designs. In embryonic prefrontal cortex, prenatal sevoflurane exposure reduced proliferation, decreased Pax6-positive progenitors, and lowered the numbers of Satb2-positive upper-layer and Tbr1-positive deep-layer immature neurons, linking progenitor disruption to abnormal cortical neurogenesis (Song et al. 2022). In a human neuron chimeric mouse model, sevoflurane impaired human neuronal migration through suppression of DVL-1/Ca^2+^ noncanonical Wnt signaling, and chemogenetic activation, DVL-1 overexpression, or targeted rTMS partially rescued migration and social phenotypes (Zhao et al., 2025). This work connects a human-cell migration defect to later behavioral consequences in an *in vivo* environment, while also emphasizing that organoid-derived human neurons can be used as a bridge between *in vitro* developmental endpoints and animal validation.

Overall, anesthetic-induced disruption of neural progenitor dynamics is best handled in organoid and bMPS studies as a trajectory-level endpoint rather than a single injury mechanism. Key measurable readouts include VZ/SVZ thickness, SOX2/PAX6/Ki67/PH3 expression, radial glial polarity, INM coherence, division-plane orientation, timing of TUJ1/MAP2/TH/SATB2/TBR1 expression, neuronal migration distance, and laminar organization. Apparent recovery of early markers should be interpreted cautiously, because transient mistiming during progenitor expansion may still reshape later cellular composition and network assembly. This progenitor-centered readout module provides a platform-relevant bridge between early tissue architecture and the mitochondrial, cell-death, synaptic, and inflammatory stress modules discussed below.

### 4.2 Mitochondrial dysfunction, neural energy metabolism, and oxidative stress

Mitochondrial dysfunction represents a major, but not universal, component of anesthetic developmental neurotoxicity. Developing neural progenitors and immature neurons have high energetic demands and relatively limited metabolic reserve, making mitochondrial integrity particularly important during neurogenesis, migration, and synaptic maturation. Across experimental models, anesthetic-associated mitochondrial abnormalities include loss of mitochondrial membrane potential, calcium dysregulation, impaired electron-transport-chain activity, altered mitochondrial quality control, cytochrome c release, and activation of downstream cell-death pathways (Sun et al., 2022; Zhang and Li, 2023; Liang et al., 2022; Ebrahimi et al., 2024). In primary hippocampal neurons, propofol reduced mitochondrial membrane potential and ATP production while increasing ROS and apoptosis-related signaling, consistent with impairment of mitochondrial respiratory-chain function (Liang et al., 2022). Volatile anesthetics may engage overlapping but non-identical pathways; for example, isoflurane exposure has been associated with mitochondrial membrane-potential disruption, increased ROS, and suppression of GPX4-related antioxidant defenses (Xia et al., 2018).

Disturbance of neural energy metabolism is closely related to, but conceptually broader than, structural mitochondrial injury. Developing neural tissues depend on coordinated glucose utilization, glycolysis, oxidative phosphorylation, creatine-dependent energy buffering, and metabolic coupling among neurons and glial cells. In propofol-exposed human cerebral organoids, reduced ATP production and decreased CKMT1B expression were accompanied by transcriptomic alterations related to mitochondrial function, synaptic activity, and inflammation (Pant et al., 2026). Region-specific metabolic responses have also been observed: dorsal and ventral forebrain organoids displayed different metabolic adaptations after propofol exposure (She et al., 2026), whereas intermittent propofol exposure in another human brain organoid model produced transcriptional and electrophysiological changes without a clear increase in lactate release (Wang et al., 2026). These observations indicate that energetic dysfunction should not be inferred from a single metabolic endpoint. ATP content, oxygen-consumption rate, glycolytic flux, glucose utilization, lactate production, mitochondrial respiratory reserve, and creatine-kinase-associated pathways should therefore be integrated with developmental and functional readouts.

Oxidative stress provides an additional link between mitochondrial dysfunction, impaired energy metabolism, and developmental injury. Excess ROS generation, impaired antioxidant defenses, lipid peroxidation, and altered iron homeostasis can damage membranes, proteins, nucleic acids, and developing neural lineages. Isoflurane-induced increases in ROS and loss of GPX4 activity support a ferroptosis-related redox mechanism (Xia et al., 2018), while prenatal and neonatal sevoflurane models implicate Nrf2-associated antioxidant disruption, iron accumulation, lipid peroxidation, oligodendrocyte ferroptosis, and subsequent hypomyelination (Song et al. 2022; Liu et al., 2025). Propofol-associated mitochondrial dysfunction can similarly increase oxidative stress (Zhang and Li, 2023; Liang et al., 2022; Ebrahimi et al., 2024), and ketamine-related developmental injury has been linked to GPX4 inhibition and inflammatory pyroptotic signaling (Bai et al., 2025).

Nitrosative stress should, however, be distinguished from both oxidative stress and physiological nitric oxide signaling. Dysregulated nitric oxide synthase activity may alter neuronal signaling, while excessive nitric oxide in an oxidative environment can favor the formation of reactive nitrogen species. Sevoflurane-associated microglial activation, for example, has been accompanied by increased iNOS expression in experimental models (Zhang et al., 2024). Nevertheless, direct evidence establishing nitrosative damage as a dominant and broadly shared mechanism of anesthetic developmental neurotoxicity remains less extensive than the evidence for ROS-mediated oxidative injury. Future organoid and bMPS studies should therefore assess oxidative and nitrosative pathways separately, combining ROS and lipid-peroxidation assays with nNOS/iNOS/eNOS expression, nitric oxide metabolites, and, where appropriate, protein-nitration markers.

### 4.3 Nitric oxide and neurotransmitter-system dysregulation with regional vulnerability

Nitric oxide (NO) signaling represents a distinct regulatory axis linking N-methyl-D-aspartate receptor activity, synaptic plasticity, neuronal development, and memory-related processes. Developmental exposure studies suggest that anesthetics can disrupt physiological NO signaling independently of, or in parallel with, overt neuronal apoptosis. In postnatal day 7 rats, a single prolonged sevoflurane exposure reduced neuronal nitric oxide synthase (nNOS) expression in the hippocampus and increased cleaved caspase-3 and neuronal loss in the CA1 and CA3 subfields (Feng et al., 2012). Importantly, this study did not demonstrate subsequent impairment in the Morris water maze, indicating that biochemical and cellular injury does not invariably translate into measurable later cognitive dysfunction.

A related neonatal isoflurane study implicated disruption of the NMDAR–PSD-95–nNOS axis in abnormal dendritic spine morphology, impaired long-term potentiation, and recognition-memory deficits. These abnormalities occurred without detectable increases in apoptosis and could be attenuated by administration of the NO donor molsidomine (Schaefer et al., 2019). These findings suggest that impaired NO-dependent synaptic plasticity may represent a sublethal pathway linking anesthetic exposure to later functional abnormalities and further illustrate why apoptosis alone is an insufficient endpoint for developmental neurotoxicity assessment.

Neurotransmitter-system disruption is likewise dependent on pharmacological target, developmental stage, and brain-region identity. GABAergic and glutamatergic systems are particularly relevant because propofol, midazolam, thiopental, and volatile anesthetics enhance GABA_A_-receptor-mediated signaling to varying degrees, whereas ketamine predominantly antagonizes NMDAR-mediated glutamatergic signaling (Hem et al., 2019; Li et al., 2025; Choudhury et al., 2021). During development, these transmitter systems contribute not only to synaptic transmission but also to progenitor behavior, neuronal migration, differentiation, and circuit assembly. Accordingly, their perturbation may produce region- and stage-specific developmental effects rather than a uniform whole-brain response.

Available evidence illustrates this regional heterogeneity. Repeated postnatal sevoflurane exposure has been associated with impaired development of GABAergic neurons and reduced neuronal synchronization in hippocampal CA2 circuitry (Wang S. et al., 2024). Maternal sevoflurane exposure can also disrupt migration of medial ganglionic eminence-derived cortical interneurons, linking altered inhibitory-circuit development to later neurological phenotypes (Liang et al., 2023). Human dorsal and ventral forebrain organoids show divergent neuronal, metabolic, and electrophysiological responses to propofol (She et al., 2026), whereas prolonged sevoflurane exposure can promote premature dopaminergic differentiation in midbrain organoids (Shang et al., 2022). Chronic neonatal midazolam exposure has additionally been associated with long-term alterations in dopamine release (Nguyen et al., 2025). Thus, hippocampal, cortical, forebrain, and midbrain circuits may exhibit distinct vulnerabilities, supporting the use of region-specific organoids and assembloids rather than a single universal brain organoid model.

### 4.4 Transcriptional dysregulation, immediate-early gene responses, and programmed cell death

Transcriptional dysregulation provides a molecular bridge between acute anesthetic exposure and delayed developmental phenotypes. Anesthetic-associated transcriptional responses encompass genes involved in neurodevelopment, synaptic maturation, mitochondrial function, inflammation, stress adaptation, and cell death, and their interpretation depends strongly on exposure timing and baseline developmental state. In human brain organoids, intermittent propofol exposure produced stage-dependent transcriptional responses, with early exposure more strongly affecting neurodevelopmental and synaptic gene programs (Wang et al., 2026). A separate propofol organoid study identified dysregulation of coding and long noncoding RNAs related to synaptic function, mitochondrial activity, and inflammation, together with reduced ATP production and altered CKMT1B expression (Pant et al., 2026). Epigenetic regulation may further contribute to persistent effects; early-life midazolam exposure has been associated with lasting changes in chromatin accessibility, impaired adult hippocampal neurogenesis, and cognitive dysfunction (Doi et al., 2021).

Immediate-early genes provide a particularly time-sensitive measure of activity-dependent transcriptional responses. In neuronal cells, clinically relevant propofol concentrations induced rapid ERK-dependent expression of c-Fos and Egr-1, whereas this response was not maintained at substantially higher concentrations (Kidambi et al., 2009). These results demonstrate that immediate-early gene responses can be both concentration- and time-dependent and should not be interpreted as nonspecific markers of toxicity. Rather, c-Fos, EGR1, ARC, and related immediate-early genes may provide dynamic readouts of neuronal activity, plasticity, and adaptive transcription when combined with developmental and functional endpoints.

Apoptosis remains one of the most extensively studied cellular outcomes of anesthetic developmental neurotoxicity. The intrinsic mitochondrial pathway is frequently implicated, involving altered BAX/BCL-2 balance, mitochondrial membrane permeabilization, cytochrome c release, and downstream caspase activation (Sun et al., 2022; Zhang and Li, 2023; Liang et al., 2022; Ebrahimi et al., 2024). In human-relevant models, sevoflurane increased cleaved caspase-3-positive cells in bMPSs and was followed by persistent abnormalities in neuronal and oligodendrocyte maturation (Li et al., 2026). Comparative neonatal animal studies further demonstrate that volatile anesthetics can produce neuroapoptotic responses whose magnitude depends on the anesthetic agent and exposure paradigm (Istaphanous et al., 2011; Kodama et al., 2011).

Recent propofol-specific studies further extend the apoptotic framework beyond mitochondrial injury. In SH-SY5Y cells and neonatal rats, nuclear FMR1-interacting protein 1 (NUFIP1)-engineered exosomes derived from human umbilical cord mesenchymal stem cells (hUMSCs) modulated propofol-associated neuronal apoptosis: NUFIP1 knockdown was associated with increased Bax and cleaved caspase-3, reduced Bcl-2, greater TUNEL-positive injury, and worse behavioral outcomes, whereas NUFIP1 overexpression produced an opposing protective profile (Sun et al., 2024). A subsequent mechanistic study linked this response to endoplasmic reticulum stress-associated apoptosis. Transcriptomic and protein analyses showed activation of PERK/GRP78/ATF4/CHOP signaling together with caspase-12, caspase-7, Bax, and caspase-3 responses after propofol exposure, while pharmacological inhibition with salubrinal attenuated these molecular changes and partially improved neurobehavioral abnormalities (Zhao et al., 2026). These findings identify proteostasis and endoplasmic reticulum stress as additional candidate components of propofol-associated programmed cell death, although the evidence derives from neuronal cell and neonatal rodent models and requires validation in human developmental 3D systems.

Apoptosis should nevertheless not be treated as the sole mechanism of developmental neurotoxicity. Ferroptosis has been associated with GPX4 suppression, lipid peroxidation, iron accumulation, and oligodendrocyte injury after isoflurane or sevoflurane exposure (Song et al. 2022; Xia et al., 2018; Liu et al., 2025); ketamine-related developmental injury has been linked to NLRP3-associated pyroptotic signaling (Bai et al., 2025); and sevoflurane-activated microglia can promote RIPK1/RIPK3/MLKL-associated neuronal necroptosis (Zhang et al., 2024). These findings support a broader programmed cell death framework in which apoptosis, ferroptosis, pyroptosis, and necroptosis are distinguished rather than inferred from a single cell-death marker. Organoid and bMPS studies should therefore combine cleaved caspase-3 and TUNEL with pathway-specific markers such as PERK/ATF4/CHOP and caspase-12 for ER stress-associated apoptosis, GPX4/SLC7A11 and lipid peroxidation for ferroptosis, NLRP3-related signaling for pyroptosis, and RIPK1/RIPK3/MLKL activation for necroptosis.

### 4.5 Synaptic, glial, neuroimmune, and network-level endpoints

Synaptic dysregulation, glial remodeling, neuroimmune activation, and circuit-level dysfunction form another set of platform-detectable endpoints in anesthetic developmental neurotoxicity. During early brain development, microglia are not only immune sentinels; they also regulate progenitor niches, synapse formation, synaptic pruning, neuronal survival, myelination, and activity-dependent circuit refinement. Therefore, anesthetic-induced microglial activation may produce lasting developmental consequences even when primary neuronal injury is modest. This endpoint domain is particularly relevant to human brain organoids, assembloids, and organ-on-chip platforms because conventional organoids often lack mature immune, vascular, and peripheral inflammatory components, which may lead to underestimation of neuroimmune amplification.

Microglia can mediate either protective or harmful responses to anesthetic agents. A recent review summarized evidence that general anesthetic agents regulate microglial proliferation, apoptosis, cytokine secretion, receptor signaling, and functional polarization, producing anti-inflammatory or pro-inflammatory effects depending on drug type, dose, exposure duration, age, and disease state (Yang et al., 2024). This duality is important for platform interpretation. For example, propofol can suppress immune-activated microglia by reducing extracellular vesicle release, thereby attenuating microglia-mediated neurotoxicity in some contexts (Liu et al., 2019). In contrast, prolonged or repeated exposure during sensitive developmental windows may shift microglia toward pro-inflammatory phenotypes, increasing IL-1β, IL-6, TNF-α, NF-κB signaling, oxidative stress, and neuron–glia injury loops (Yang et al., 2024). Ketamine shows a similarly context-dependent profile: at anesthetic doses during neurodevelopmental windows, it has been linked to inflammation, autophagy, apoptosis, and ROS generation, whereas subanesthetic exposure can activate neurotrophic signaling and produce neuroprotective effects (Choudhury et al., 2021). These findings indicate that microglial readouts in organoid systems should be interpreted in relation to exposure paradigm, maturation stage, and immune-cell state.

Sevoflurane-related evidence highlights microglia as active mediators of neuronal injury and synaptic pathology. In BV2 microglia, sevoflurane promoted M1 polarization and activated the cGAS/STING pathway; microglial STING knockdown reduced amoeboid morphology, TNF-α release, iNOS, CD16, and CD32 expression, and also decreased neuronal calcium overload and RIPK1/RIPK3/p-MLKL-dependent necroptosis in conditioned neuronal cultures (Zhang et al., 2024). These findings suggest that anesthetic exposure can convert microglia into upstream drivers of neuronal necroptosis, linking innate immune sensing to calcium dysregulation and inflammatory cell death. For human 3D neural platforms, this mechanism can be translated into measurable endpoints such as microglial morphology, cytokine release, cGAS/STING activation, neuronal calcium dynamics, and necroptosis-related markers.

Complement-mediated synaptic elimination provides a second readout layer. In a prolonged sevoflurane anesthesia model, anesthesia activated the NF-κB inflammatory pathway, enhanced microglial migration, activation, and phagocytosis, increased hippocampal C1qa and C3, and promoted C1qa tagging of synapses (Xu et al., 2023). This “Eat Me” complement cascade led to microglial engulfment of synapses, loss of dendritic spines and synaptic proteins, reduced neuronal excitability, cognitive dysfunction, and anxiety-like behaviors (Xu et al., 2023). Microglial depletion, neuroinflammatory inhibition, or C1q neutralization rescued synaptic loss and behavioral deficits (Xu et al., 2023). These results support the inclusion of complement factors, synaptic puncta, dendritic spine density, and microglial engulfment assays when organoid or chip models are used to study whether anesthetic exposure alters activity-dependent synaptic refinement.

Developmental synaptic and network phenotypes are also evident after early-life exposure. Repeated postnatal sevoflurane exposure impaired adult social novelty recognition and reduced CA2 neuronal spike synchronization, gamma oscillation power, and spike-phase coupling between GABAergic spiking and gamma oscillations during social interaction (Wang S. et al., 2024). These electrophysiological abnormalities were accompanied by reduced density and dendritic complexity of CA2 GABAergic neurons and decreased expression of transcription factors required for GABAergic neuronal development (Wang S. et al., 2024). Although such region-specific behavioral circuits cannot be fully reproduced in isolated organoids, the relevant endpoint logic can be modeled through GABAergic and glutamatergic synaptic markers, calcium imaging, MEA recording, oscillatory activity, and network synchronization.

Human organoid-based systems are beginning to capture this neuroimmune–synaptic axis more directly. An ICU patient-on-a-chip model incorporating hiPSC-derived mast cells, cerebral organoids, and a BBB-like endothelial compartment showed that prolonged propofol or midazolam administration produced distinct neuroinflammatory and neurotoxic signatures (Saglam-Metiner et al., 2024a). Propofol increased mast-cell CD40 and TNF-α, microglial AIF1, macroglial GFAP/S100B/OLIG2/MBP, and organoid inflammatory markers including NOS2, CD80, CD40, CD68, IL6, and TNF-α (Saglam-Metiner et al., 2024a). Midazolam more strongly disrupted BBB permeability, reduced TEER values, increased CD11b^+^ microglia, altered GFAP/DLG4/GJB1 and GABA_A_/NMDAR1-related identities, and promoted glutamate-related neurotoxicity and apoptotic zones (Saglam-Metiner et al., 2024a). These findings show that BBB integrity, mast-cell activation, microglial reactivity, astroglial responses, and neuronal receptor programs can interact to shape sedative-induced injury. They also illustrate why standard cerebral organoids without immune and vascular components may miss inflammatory amplification loops that become visible in BBB- or immune-integrated chip systems.

Systemic immune-metabolic mechanisms further define the limits of isolated brain platforms. Repeated neonatal sevoflurane exposure altered the gut microbiota–metabolite–brain axis and impaired social behavior and synaptic development; fecal microbiota transplantation and bile-acid regulation attenuated these social and synaptic deficits (Zhao et al., 2024). Although such mechanisms are difficult to model in isolated organoids, they emphasize that developmental anesthetic neurotoxicity may involve both central neural tissue responses and peripheral immune-metabolic signals. This limitation supports the use of organoid and chip platforms as reductionist systems for testing defined interfaces, rather than complete replacements for animal or clinical studies.

**FIGURE 2:**
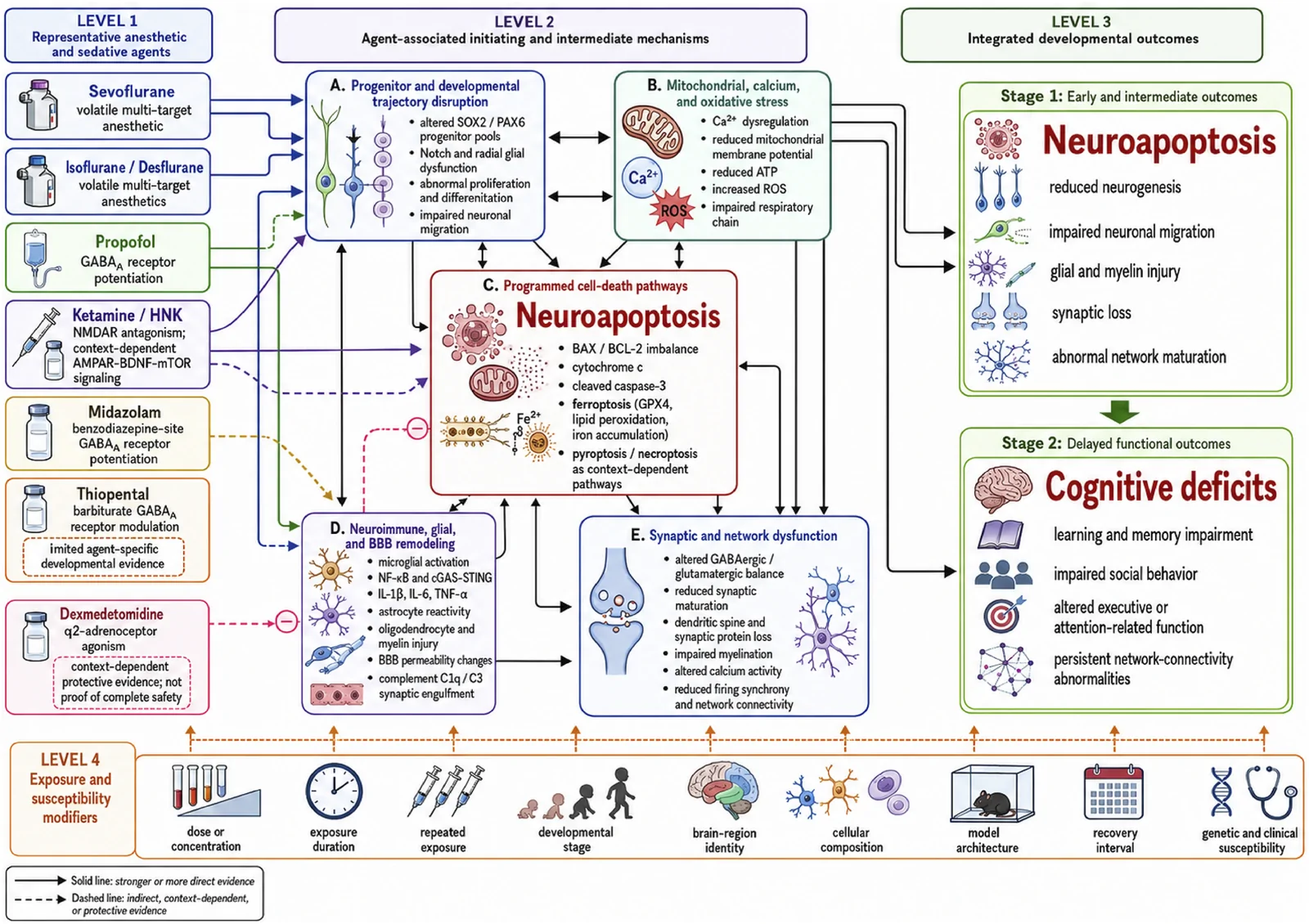
Agent-specific and convergent progression of anesthetic developmental neurotoxicity toward neuroapoptosis and cognitive dysfunction. Different anesthetic and sedative agents initiate partially overlapping but non-identical responses through their predominant receptor and ion-channel actions. Depending on dose or concentration, exposure duration and repetition, developmental stage, brain-region identity, cellular composition, model architecture, and host susceptibility, these initiating events may disturb progenitor maintenance and neuronal migration, mitochondrial and calcium homeostasis, redox balance, neuroimmune and glial signaling, blood–brain barrier integrity, synaptic maturation, myelination, and network organization. These interacting pathways may converge on programmed neuronal loss, including neuroapoptosis, and on persistent circuit dysfunction that is associated in preclinical models with later impairments in learning, memory, social behavior, and other cognitive domains. Solid connections indicate comparatively stronger or more direct evidence, whereas dashed connections indicate limited, context-dependent, or potentially protective relationships. Not every pathway is engaged by every agent, and organoid-derived cellular phenotypes should not be interpreted as direct evidence of clinical cognitive impairment. AMPAR, α-amino-3-hydroxy-5-methyl-4-isoxazolepropionic acid receptor; BBB, blood–brain barrier; BDNF, brain-derived neurotrophic factor; GABA_A_​, γ-aminobutyric acid type A receptor; HNK, hydroxynorketamine; NMDAR, N-methyl-D-aspartate receptor; ROS, reactive oxygen species.

Together, these findings indicate that anesthetic developmental neurotoxicity converges on synaptic, glial, neuroimmune, and network-level endpoints. Relevant platform readouts include microglial identity and morphology, cytokine secretion, NF-κB and cGAS/STING signaling, complement factors C1q/C3, synaptic engulfment, astrocyte reactivity, BBB permeability, GABAergic and glutamatergic synaptic markers, calcium dynamics, oscillatory activity, and network synchronization. Integrating microglia, astrocytes, oligodendrocytes, endothelial cells, and selected peripheral immune components into organoid or organ-on-chip platforms will be essential for determining whether anesthetic exposure mainly delays maturation or actively remodels human neural circuits through maladaptive neuroinflammatory pruning. Across anesthetic and sedative classes, developmental neurotoxicity is best represented as a staged and context-dependent sequence rather than as a single uniform lesion. Under vulnerable exposure conditions, drug-specific receptor actions may initiate progenitor disruption, calcium and mitochondrial dysregulation, oxidative stress, neuroinflammatory amplification, and synaptic or glial injury. These intermediate events may converge on programmed neuronal loss, including neuroapoptosis, and persistent disruption of circuit maturation. Human brain organoid and bMPS platforms can capture many of these cellular and network-level intermediates, whereas animal models currently provide the principal evidence linking early injury to later deficits in learning, memory, social behavior, and other cognitive domains (Li et al., 2026; Liu et al., 2025; Wang S. et al., 2024; Doi et al., 2021; Istaphanous et al., 2011; Xu et al., 2023; Dong and Anand, 2013). The agent-specific routes and their convergence on neuroapoptosis and cognitive dysfunction are summarized in Figure 2.

## 5 Biological and engineering variables shaping divergent anesthetic responses

### 5.1 Drug class, pharmacodynamics, developmental toxicodynamics, and exposure paradigm

Divergent anesthetic responses should be interpreted not only as drug-specific toxicity profiles, but also as a consequence of how each drug is delivered, maintained, measured, and removed during washout in organoid and organ-on-chip platforms. Sevoflurane, propofol, ketamine, and midazolam can all affect apoptosis, mitochondrial homeostasis, synaptic maturation, and neuroinflammatory signaling, but their primary pharmacology, exposure route, exposure stability, dose or concentration, and washout period can bias the direction, timing, and cellular distribution of platform readouts. To distinguish intended drug effects from developmental injury pathways, pharmacodynamics is used here to describe the receptor-, circuit-, and system-level actions underlying hypnosis, amnesia, immobility, analgesia, or sedation, whereas developmental toxicodynamics refers to the cellular and tissue responses produced when exposure occurs during vulnerable neurodevelopmental windows. These responses may include altered progenitor behavior, mitochondrial and oxidative stress, programmed cell death, synaptic or glial remodeling, and network dysfunction. Importantly, the toxicodynamic profiles summarized below are exposure-, stage-, and model-dependent and should not be interpreted as a clinical ranking of comparative anesthetic safety (Hem et al., 2019; Zhang and Li, 2023). Representative pharmacological mechanisms, principal pharmacodynamic effects, and developmental toxicodynamic profiles of commonly used general anesthetics and sedatives are summarized in Table 2.

**TABLE 2:** Pharmacological and developmental toxicodynamic profiles of representative general anesthetics and sedatives.

Agent and class	Predominant mechanism of action	Principal pharmacodynamic effects	Developmental toxicodynamic profile	Representative evidence
Sevoflurane; volatile inhalational anesthetic	Multi-target modulation of ligand-gated and voltage-gated ion channels, including potentiation of inhibitory GABAA and glycine signaling and modulation of excitatory and potassium-channel activity	Dose-dependent hypnosis, amnesia, immobility, and suppression of neuronal network activity; suitable for inhalational induction and maintenance	Exposure- and stage-dependent disruption of ventricular zone-like organization, radial glial interkinetic nuclear migration and Notch signaling; mitochondrial and calcium dysregulation, oxidative stress, apoptosis or ferroptosis, altered synaptic maturation, glial remodeling, and network dysfunction have also been reported	(Hem et al., 2019; Lee et al., 2022; Jiang et al., 2021; Liu et al., 2025)
Isoflurane; volatile inhalational anesthetic	Multi-target volatile anesthetic acting on GABAA, glycine, nicotinic acetylcholine, potassium, and other ion-channel systems	Hypnosis, amnesia, immobility, reduced neuronal excitability, and dose-dependent cardiorespiratory depression	Preclinical developmental exposure has been associated with mitochondrial membrane-potential loss, increased ROS, reduced GPX4 signaling, neuroapoptosis or ferroptotic injury, and later cognitive or behavioral abnormalities	(Xia et al., 2018; Istaphanous et al., 2011)
Desflurane; volatile inhalational anesthetic	Multi-target volatile anesthetic with actions on inhibitory and excitatory ion channels	Rapidly titratable hypnosis, amnesia, and immobility because of low blood-gas solubility	Comparative neonatal mouse findings are not fully consistent: one study reported broadly similar neuroapoptotic profiles among equipotent volatile anesthetics, whereas another found more extensive neuroapoptosis and impaired working memory after desflurane exposure	(Istaphanous et al., 2011; Kodama et al., 2011)
Propofol; intravenous general anesthetic and sedative	Predominantly positive allosteric modulation of GABAA receptors, with additional actions on ion channels and neuronal networks	Rapid-onset hypnosis and amnesia with rapid recovery; anticonvulsant and sedative effects but little intrinsic analgesia	Calcium overload, mitochondrial respiratory-chain and bioenergetic impairment, reduced ATP and mitochondrial membrane potential, increased ROS, autophagic and endoplasmic-reticulum stress, apoptosis and other programmed-cell-death pathways, altered neurotrophin signaling, synaptic dysregulation, inflammation, and noncoding RNA changes	(Zhang and Li, 2023; Ebrahimi et al., 2024; She et al., 2023)
Ketamine; intravenous dissociative anesthetic	Noncompetitive N-methyl-D-aspartate receptor antagonism; downstream AMPA receptor, BDNF, and mTOR signaling may become important under subanesthetic exposure conditions	Dissociative anesthesia, analgesia, amnesia, and altered cortical-subcortical connectivity	Developmental effects are strongly concentration- and stage-dependent. Anesthetic or repeated exposure has been associated with neuronal apoptosis, impaired progenitor proliferation, altered differentiation, mitochondrial and oxidative stress, inflammation, autophagy or pyroptosis; lower or subanesthetic exposure can instead engage neurotrophic and plasticity-related pathways	(Du et al., 2023; Dong et al., 2012; Choudhury et al., 2021; Dong and Anand, 2013)
Thiopental; barbiturate intravenous anesthetic	Positive allosteric modulation of GABAA receptors and prolongation of chloride-channel opening; direct receptor activation can occur at higher concentrations	Rapid hypnosis and anticonvulsant activity, without clinically meaningful analgesia; dose-dependent respiratory and cardiovascular depression	Agent-specific developmental toxicodynamic evidence is relatively limited. In one neonatal mouse combination study, thiopental did not enhance sevoflurane-induced neuroapoptosis, whereas propofol did. This result does not establish absence of thiopental neurotoxicity and should not be generalized beyond the tested exposure paradigm	(Tagawa et al., 2014)
Midazolam; benzodiazepine sedative	Positive allosteric modulation of GABAA receptors through the benzodiazepine-binding site	Anxiolysis, sedation, anterograde amnesia, anticonvulsant activity, and hypnosis, with little intrinsic analgesia	Prolonged or repeated early-life exposure has been associated with persistent chromatin-accessibility changes, impaired hippocampal neurogenesis, abnormal mTOR signaling and neural-circuit maturation, inflammation, glial and receptor remodeling, BBB disruption, and apoptosis in model-dependent settings	(Saglam-Metiner et al., 2024a; Nguyen et al., 2025; Doi et al., 2021)
Dexmedetomidine; α2-adrenoceptor agonist sedative	Highly selective activation of central α2-adrenoceptors, particularly within the locus coeruleus and endogenous sleep-arousal circuits	Arousable sleep-like sedation, anxiolysis, sympatholysis, and analgesic- and anesthetic-sparing effects, with relatively limited respiratory depression	Direct developmental injury evidence is limited, whereas many preclinical studies report attenuation of volatile-anesthetic-induced apoptosis, inflammation, and later cognitive abnormalities. These protective findings remain exposure- and model-dependent and should not be interpreted as proof of complete developmental safety	(Weerink et al., 2017; Sanders et al., 2009)

Sevoflurane represents a volatile-anesthetic exposure pattern in which gas delivery, exposure duration, and actual tissue concentration are especially important for interpretation. Developmental toxicity has been linked to receptor imbalance, calcium dysregulation, neuroapoptosis, tau phosphorylation, neuroendocrine alteration, and neuroinflammation (Sun et al., 2022). In human cerebral organoids and fetal cortical models, sevoflurane exposure has been associated with transient disruption of ventricular zone-like structures, accelerated neuronal marker expression, and impaired radial glial interkinetic nuclear migration through Notch-related mechanisms (Lee et al., 2022; Jiang et al., 2021). Thus, sevoflurane-sensitive organoid assays are particularly useful for testing whether inhalational exposure alters progenitor-zone organization, radial glial behavior, and early differentiation before overt tissue loss is detectable. For cross-platform comparison, however, nominal gas concentration should be interpreted alongside exposure duration, culture format, and recovery interval.

Propofol produces a different readout profile because it is commonly modeled as liquid-phase exposure and is often associated with mitochondrial and metabolic vulnerability. Although it acts mainly through GABA_A_ receptor potentiation, preclinical studies link propofol developmental neurotoxicity to calcium overload, abnormal autophagy, mitochondrial dysfunction, endoplasmic reticulum stress, neurotrophin disruption, synaptic dysregulation and noncoding RNA changes (Zhang and Li, 2023). A representative *in vivo* study showed that repeated propofol exposure in PND7 rats increased neuronal injury, enhanced calpain activity, downregulated TRPC6, and produced later learning and memory deficits; TRPC6 activation or calpain inhibition attenuated these effects (She et al., 2023). In organoid or bMPS studies, propofol-sensitive endpoints should therefore include ATP production, mitochondrial membrane potential, calcium-calpain signaling, synaptic proteins, and recovery-phase network activity, rather than apoptosis alone. Device material, medium volume, repeated dosing, and washout timing may also influence the observed response profile.

Ketamine illustrates how dose, developmental timing, and endpoint selection can change interpretation. At anesthetic or repeated developmental exposures, NMDAR antagonism can impair proliferation, enhance oxidative stress, and promote apoptosis, autophagy, inflammation, or pyroptosis. Recent neonatal rat data further implicate GPX4 inhibition and NLRP3-mediated pyroptosis in ketamine-related hippocampal injury and cognitive impairment (Bai et al., 2025). However, ketamine and its metabolite HNK can also activate AMPAR-dependent neurotrophic signaling, including BDNF- and mTOR-related pathways, especially under subanesthetic antidepressant-like paradigms (Choudhury et al., 2021). Therefore, ketamine/HNK should not be classified as simply neurotoxic or neuroprotective. In human 3D neural platforms, its readout profile should be interpreted according to concentration range, exposure window, receptor-subunit composition, synaptic versus extrasynaptic NMDAR signaling, and whether the assay captures acute stress or adaptive plasticity.

Midazolam adds a sedative-specific exposure paradigm because it is often relevant to prolonged neonatal or intensive-care sedation rather than brief surgical anesthesia. As a benzodiazepine GABA_A_ receptor modulator, chronic neonatal midazolam exposure in rats altered early physical development, reduced adult dopamine release, increased inflammatory cytokines and growth factors, and produced stage-dependent anxiety-like and social-interaction changes (Nguyen et al., 2025). In bioengineered BBB–organoid–immune platforms, propofol and midazolam produced overlapping but distinguishable endothelial, mast-cell, glial, cytokine, receptor, and apoptotic responses (Saglam-Metiner et al., 2024a). These findings suggest that benzodiazepine-related platform readouts may reflect prolonged remodeling of inhibitory signaling, neuroimmune tone, BBB-associated interactions, and glial–neuronal communication, rather than acute neuronal loss alone.

Together, these examples show that divergent anesthetic responses arise from both pharmacology and platform-defined exposure conditions. Each anesthetic should be profiled across receptor signaling, mitochondrial stress, progenitor dynamics, synaptic maturation, glial activation, BBB or immune responses, and network activity, while reporting dose or concentration, delivery route, exposure duration, repetition, and recovery interval. This exposure-aware profiling can explain why agents that converge on shared developmental stress modules nevertheless produce different readout profiles across organoids, bMPSs, organ-on-chip systems, animal models, and clinical exposure scenarios.

### 5.2 Combination exposure and potentiation by concomitant neuroactive drugs

Developmental neurotoxicity may be influenced not only by the properties of an individual anesthetic but also by concomitant exposure to other anesthetic, sedative, or neuroactive drugs. This issue is clinically relevant because general anesthesia and intensive care sedation frequently involve multidrug regimens, and vulnerable neonates or infants may simultaneously receive other central nervous system-active medications. Experimental evidence suggests that combinations engaging complementary inhibitory and excitatory neurotransmitter systems can, under some exposure conditions, produce greater neurodevelopmental injury than individual agents alone.

One of the clearest examples involves simultaneous enhancement of GABA_A_-receptor-mediated inhibition and blockade of N-methyl-D-aspartate receptors. In neonatal mice, coadministration of ketamine with either propofol or thiopental produced greater apoptotic neurodegeneration and persistent behavioral abnormalities than the relatively limited effects observed with several of the individual low-dose agents (Fredriksson et al., 2007). Similarly, exposure of infant rats to a combination of midazolam, nitrous oxide, and isoflurane produced widespread apoptotic neurodegeneration, impaired hippocampal synaptic function, and persistent learning and memory deficits (Jevtovic-Todorovic et al., 2003). These findings support the concept that simultaneous perturbation of excitatory and inhibitory signaling during critical developmental windows may amplify injury, although the relative contribution of each component depends on dose and exposure duration.

Potentiation is not, however, uniform across all drug combinations. In neonatal mice, sevoflurane combined with propofol produced more robust neuroapoptosis than sevoflurane alone, whereas thiopental did not produce the same potentiating effect under the tested conditions (Tagawa et al., 2014). This agent-specific difference cautions against assuming that all anesthetic combinations have equivalent toxicodynamic interactions. Combination effects may reflect receptor-level convergence, cumulative suppression of neuronal activity, mitochondrial or metabolic stress, prolonged effective exposure, or interactions among multiple injury pathways.

Other neuroactive co-medications may also modify anesthetic neurotoxicity. Caffeine is particularly relevant to neonatal medicine because it is commonly used in premature infants for apnea of prematurity. In infant mice, caffeine alone produced relatively weak neuroapoptotic effects but markedly potentiated neuroapoptosis when combined with several neuroactive agents, including midazolam, diazepam, ketamine, and isoflurane; selected combination exposures were also associated with more pronounced long-term behavioral abnormalities than either drug alone (Yuede et al., 2013). These findings illustrate that drugs outside conventional anesthetic classes may alter developmental vulnerability and should be considered when modeling clinically relevant exposure scenarios.

Nevertheless, these observations are derived predominantly from animal models and should not be interpreted as evidence that multidrug anesthesia is intrinsically more neurotoxic in children. In clinical practice, balanced anesthesia may permit lower doses of individual agents, and surgery, pain, inflammation, physiological instability, and underlying disease further complicate attribution of neurodevelopmental outcomes to specific drug combinations. For organoid and brain microphysiological studies, combination-exposure designs should therefore compare single agents with clinically plausible combinations while controlling total exposure burden, concentration, duration, developmental stage, and recovery interval. Such studies may help distinguish additive, synergistic, or antagonistic toxicodynamic interactions and identify whether specific combinations preferentially amplify neuroapoptosis, mitochondrial stress, transcriptional responses, or delayed network dysfunction.

### 5.3 Neuroprotective and cognition-preserving pharmacological strategies

Preclinical studies have identified several pharmacological interventions that can attenuate specific components of anesthetic developmental neurotoxicity. These strategies do not act through a single common pathway; instead, reported protective effects involve suppression of apoptosis and neuroinflammation, preservation of mitochondrial and redox homeostasis, restoration of neurotrophic signaling, and stabilization of synaptic or glial development. Importantly, most evidence remains preclinical and strongly dependent on anesthetic agent, developmental stage, dose, timing of administration, and experimental model. No pharmacological intervention has yet been established as a standard clinical prophylaxis against anesthetic developmental neurotoxicity in infants or children. Accordingly, these compounds are better considered mechanistic rescue probes and candidate protective strategies rather than clinically validated neuroprotective treatments (Johnson et al., 2019; Bleese et al., 2023).

Dexmedetomidine is among the most extensively investigated candidates. In neonatal rats, dexmedetomidine attenuated isoflurane-induced neurocognitive impairment (Sanders et al., 2009). Related developmental models have shown protection against sevoflurane-associated neuroapoptosis, neuroinflammation, oxidative stress, and later cognitive abnormalities through α2-adrenoceptor-dependent mechanisms (Zhang et al., 2021). More recently, dexmedetomidine administered during maternal sevoflurane exposure was reported to mitigate offspring hippocampal apoptosis, abnormalities in BDNF receptor signaling, dendritic spine loss, and later cognitive deficits (Yu et al., 2025). These findings support anti-apoptotic, anti-inflammatory, redox-regulatory, and neurotrophic mechanisms, but they remain insufficient to establish developmental neuroprotection in pediatric clinical practice.

Melatonin represents another candidate with convergent mitochondrial, antioxidant, and synaptic effects. In neonatal mice repeatedly exposed to sevoflurane, melatonin suppressed hippocampal reactive oxygen species and cyclophilin D expression, preserved mitochondrial membrane potential and synaptic protein expression in parvalbumin-positive neurons, and attenuated subsequent cognitive deficits (Zou et al., 2023). Other mechanistically distinct rescue strategies further illustrate the range of potentially modifiable injury pathways. In infant non-human primates, lithium coadministration prevented acute isoflurane-induced neuronal apoptosis and substantially reduced oligodendroglial apoptosis (Noguchi et al., 2016). Erythropoietin pretreatment also reduced sevoflurane-associated neuronal degeneration and long-term memory deficits in neonatal rats (Goyagi, 2019). At the mitochondrial level, coenzyme Q10 attenuated propofol-associated loss of ATP and mitochondrial membrane potential and oxidative/apoptotic injury in primary hippocampal neurons (Liang et al., 2022). Engineered extracellular-vesicle strategies are also emerging as mechanistic rescue approaches; NUFIP1-overexpressing hUMSC-derived exosomes attenuated propofol-associated apoptotic and neurobehavioral abnormalities in neonatal models, with subsequent work implicating ER-stress-associated apoptotic signaling as a modifiable pathway (Sun et al., 2024; Zhao et al., 2026).

Evidence for classical nootropic drugs as countermeasures against anesthetic developmental neurotoxicity is considerably less developed than that for the neuroprotective strategies described above. Therefore, cognition-enhancing agents should not be assumed to prevent developmental neural injury solely because they improve behavioral performance in other neurological settings. A more informative approach is to evaluate candidate protective compounds in organoids and bMPSs using paired injury-and-rescue designs. Such studies could determine whether an intervention restores the multidimensional injury fingerprint, including progenitor and lineage markers, mitochondrial function, ROS, programmed cell death, synaptic maturation, glial responses, and network activity, rather than merely suppressing a single endpoint such as cleaved caspase-3. Because candidate neuroprotective agents may themselves alter neuronal differentiation or activity, their independent developmental effects should also be evaluated. This framework could enable human-relevant prioritization of protective strategies before animal and clinical validation.

### 5.4 Developmental age/timing, brain-region identity, and lineage composition

Developmental timing and brain-region identity are major variables shaping divergent anesthetic responses in organoid and related platform studies. The same anesthetic may affect neural progenitor behavior during early corticogenesis, neuronal migration during fetal circuit assembly, synaptogenesis during perinatal maturation, or glial and network functions during later development. Therefore, developmental stage determines not only the magnitude of the response, but also which endpoint should be prioritized. In platform design, early cortical organoids, forebrain assembloids, midbrain organoids, oligodendrocyte-enriched systems, and BBB- or immune-integrated models answer different questions and should not be treated as interchangeable models of whole-brain risk.

At early developmental stages, anesthetic exposure is more likely to affect neural progenitor organization, neuroepithelial integrity, and the timing of neuronal differentiation. In hESC-derived cerebral organoids exposed to sevoflurane during a first-trimester-equivalent window, intermediate sevoflurane exposure did not persistently reduce organoid size, but transiently decreased SOX2^+^/N-cadherin^+^ ventricular zone-like structures and increased early neuronal markers such as TUJ1 and MAP2, followed by later normalization of these markers and cortical architecture (Lee et al., 2022). This pattern suggests that early cortical organoids are particularly useful for detecting developmental trajectory shifts rather than only irreversible tissue loss. However, apparent marker recovery should be interpreted cautiously, because gross normalization may still conceal changes in lineage timing, neuronal subtype composition, or later network competence.

During mid-gestational stages, migration and regional circuit assembly become more relevant endpoints. Maternal sevoflurane exposure at embryonic day 14.5 in mice disrupted the ordered tangential migration of MGE-derived cortical interneurons, downregulated CXCL12/CXCR4 signaling, and produced adolescent phenotypes including increased seizure susceptibility and anxiety- or depression-like behaviors (Liang et al., 2023). These effects were evident in adolescence but did not persist into adulthood, indicating that anesthetic-induced developmental alterations may be delayed, stage-restricted, or compensated over time. For organoid-based modeling, this supports the use of dorsal–ventral forebrain assembloids or migration-focused platforms when the research question concerns interneuron positioning and excitatory–inhibitory balance.

Later neonatal exposure may preferentially affect glial maturation, myelination, and neuron–glia metabolic coupling. Repeated neonatal sevoflurane exposure from P6 to P8 produced later hypomyelination and neurological impairment through DHHC5 downregulation, reduced TfR1 palmitoylation, enhanced TfR1 endocytosis, iron accumulation, and ferroptosis in oligodendrocytes (Liu et al., 2025). Because TfR1 expression in oligodendrocyte lineage cells is transient during early postnatal life, this mechanism illustrates a temporally restricted vulnerability window. It also indicates that developmental neurotoxicity may arise from non-neuronal lineages, with oligodendrocyte ferroptosis secondarily promoting neuronal injury in regions such as the prefrontal cortex and hippocampal CA1 (Liu et al., 2025). Accordingly, oligodendrocyte-rich or myelination-competent platforms are better suited than early cortical organoids for studying glial and white-matter-related endpoints.

Brain-region divergence further emphasizes the need for model selection based on the biological question. Cortical organoids emphasize ventricular zone dynamics, radial glia, neuronal differentiation, and migration; forebrain assembloids are better suited for excitatory–inhibitory balance and interneuron migration; hippocampal or cortical–hippocampal readouts may be more sensitive to memory-related synaptic plasticity; white-matter-enriched systems reveal oligodendrocyte and myelination phenotypes; and midbrain organoids capture dopaminergic lineage vulnerability. Region-specific organoid studies support this principle: dorsal and ventral forebrain organoids can show different neuronal, metabolic, and electrophysiological responses to propofol (She et al., 2026), intermittent propofol exposure alters activity and neurodevelopmental gene programs after early but not later exposure (Wang et al., 2026), prolonged sevoflurane can promote premature dopaminergic differentiation in midbrain organoids (Shang et al., 2022), and chronic high-concentration HNK exposure can suppress neural precursor expansion and alter radial glial division orientation in cerebral organoids (Du et al., 2023). Human neuronal chimeric models further show that sevoflurane can impair human neuronal migration through DVL-1/Ca^2+^-related signaling *in vivo* (Zhao et al., 2025).

Together, developmental-stage and brain-region divergence argue against a single universal toxicity endpoint or a single universal organoid model. Anesthetic responses should instead be mapped onto a platform-selection matrix linking exposure timing, regional identity, lineage composition, and functional maturation state. In organoid-based studies, early NPC markers, migration assays, synaptic proteins, glial maturation, myelination, calcium dynamics, and electrophysiological network activity should be selected according to the modeled developmental window and brain-region identity. This approach makes divergent responses more interpretable and supports cross-platform comparison rather than overgeneralization from one model system.

### 5.5 Model-dependent divergence

Model-dependent divergence is a major source of inconsistency in anesthetic developmental neurotoxicity research. Different experimental systems capture different levels of biological organization, and therefore may yield different results even when the same anesthetic, dose, or exposure window is tested. These discrepancies should not be treated simply as contradictions. Instead, they indicate that each model answers a different question and should be placed within a benchmarking framework that links cell-autonomous mechanisms, human developmental trajectories, immune–vascular exposure biology, systemic validation, and clinical relevance.

Two-dimensional neural cultures remain useful for defining direct cellular toxicity, receptor signaling, calcium overload, mitochondrial dysfunction, oxidative stress, and apoptotic thresholds under highly controlled conditions. However, they cannot reproduce three-dimensional tissue architecture, radial organization, multicellular lineage interactions, extracellular matrix constraints, or emerging circuit activity. As a result, 2D models may overemphasize acute cell-autonomous injury while underestimating developmental trajectory shifts, regional patterning defects, and delayed network consequences. This limitation is important because preclinical intervention studies often identify mechanisms such as ROS-mediated stress, growth and nutrient signaling, and direct neuronal modulation, but their relative importance may change in more complex systems (Johnson et al., 2019).

Three-dimensional brain organoids partly overcome these limitations by preserving human genetic background, neural progenitor zones, radial glial-like structures, neuronal differentiation, early glial development, and spontaneous activity. They are well suited to detecting human developmental perturbations that may not be captured in simple cultures or rodents. However, organoid outputs are shaped by model architecture, protocol, matrix environment, tissue size, batch variability, incomplete maturation, limited vascularization, and imperfect microglial or immune representation. Organoids also lack systemic metabolism, maternal–fetal physiology, ventilation, inflammation, pain, surgery, and behavioral outcomes. Therefore, an organoid phenotype should be interpreted as a human-relevant mechanistic signal, not as a direct prediction of postoperative IQ, behavior, or school performance.

Brain microphysiological and organ-on-chip systems introduce another benchmarking layer because they incorporate fluid flow, BBB-like barriers, compartmentalized exposure, immune crosstalk, matrix-defined tissue environments, and longitudinal sampling. In an ICU patient-on-a-chip platform, propofol and midazolam produced partly overlapping but distinguishable effects when cerebral organoids were combined with hiPSC-derived mast cells and a BBB-like endothelial layer. Propofol preferentially enhanced TNF-α-related proinflammatory responses, glial marker expression, and altered GABA_A_/NMDAR-related identities, whereas midazolam more strongly disrupted BBB permeability, reduced TEER, increased CD203c^+^ mast-cell reactivation, elevated glutamate release, and expanded apoptotic zones (Saglam-Metiner et al., 2024a). The response differed from cerebral organoids cultured without BBB and mast-cell components, indicating that immune–vascular context can amplify or redirect apparent neurotoxicity (Saglam-Metiner et al., 2024a). These findings show why chip material, flow profile, barrier maturity, immune-cell inclusion, matrix composition, exposure duration, and recovery interval need to be reported and compared across platforms.

Animal models remain indispensable for systemic physiology, pharmacokinetics, maternal–fetal interaction, behavior, and long-term outcome assessment. Rodents and non-human primates have consistently demonstrated anesthetic-associated neuronal injury and neurobehavioral impairment, especially after repeated, prolonged, or high-dose exposure (Bleese et al., 2023). Yet species differences in cortical development, progenitor composition, synaptogenesis, glial maturation, anesthetic metabolism, and behavioral assays limit direct translation. Some preclinical paradigms also use supraclinical exposure durations or insufficient physiological monitoring, which can confound interpretation (Bleese et al., 2023). Human neuronal chimeric animals partially bridge this gap by placing human neurons into an *in vivo* environment, but they remain constrained by host species physiology and circuit integration (Zhao et al., 2025).

Clinical samples and epidemiological cohorts represent the final translational layer, but they introduce additional sources of divergence, including surgery, inflammation, prematurity, underlying disease, socioeconomic factors, repeated procedures, and perioperative instability. Therefore, clinical null findings after a short single exposure do not invalidate organoid or animal injury signals, just as strong preclinical toxicity does not automatically prove clinical harm. A coherent framework should align each model with its appropriate question: 2D cultures for cell-autonomous mechanisms, organoids for human developmental trajectories, bMPSs for standardized 3D neural responses, organ-on-chip systems for immune–vascular exposure biology, animals for systemic and behavioral validation, and clinical studies for population-level associations. Only through cross-platform validation can anesthetic developmental neurotoxicity be interpreted as a graded, context-dependent response rather than a single transferable endpoint. The major biological and engineering variables shaping divergent responses are summarized in Table 3.

**TABLE 3:** Sources of divergent anesthetic responses across drugs, developmental stages, brain regions, and models.

Divergence source	Variables/contrasts	Likely affected layer	Representative readouts	Model-design implication	Refs
Drug class	Sevoflurane, propofol, ketamine/HNK, midazolam	Different receptor entry points bias shared stress networks: progenitor/Notch, mitochondria-calcium, NMDAR/AMPAR plasticity, or inhibitory-neuroimmune remodeling	Receptor signaling, calcium-calpain, ATP/ROS, synaptic markers, cytokines	Profile drugs as multidimensional fingerprints rather than binary safe/toxic categories	(Lee et al., 2022; Jiang et al., 2021; Saglam-Metiner et al., 2024a; Zhang and Li, 2023; Nguyen et al., 2025; She et al., 2023)
Dose, duration, repetition	Single short exposure *versus* prolonged, repeated, or chronic exposure	Higher exposure burden may shift transient adaptation toward persistent stress, glial injury, or circuit dysfunction	Dose-response, recovery interval, apoptosis/ferroptosis, MEA activity	Brief null findings should not be generalized to prolonged or repeated exposure	(McCann et al., 2019; Xin et al., 2025; Shang et al., 2022; Liu et al., 2025; Liang et al., 2023)
Developmental stage	Early corticogenesis, mid-gestational migration, neonatal synaptogenesis/myelination	Dominant vulnerability changes from progenitors to migration, synapses, oligodendrocytes, and network refinement	SOX2/PAX6/Ki67, INM, migration, synapses, myelin, synchrony	Match endpoint panels to biological stage, not just culture day	(Lee et al., 2022; Jiang et al., 2021; Liu et al., 2025; Liang et al., 2023)
Brain-region identity	Dorsal/ventral forebrain, midbrain, hippocampus/CA2, corpus callosum	Regional identity determines lineage composition, neurotransmitter phenotype, metabolism, and circuit sensitivity	Regional markers, TH/GABAergic markers, CA2 oscillations, myelin	Use region-specific organoids or assembloids when regional vulnerability is central	(She et al., 2026; Shang et al., 2022; Liu et al., 2025; Wang et al., 2024a)
Cell lineage	NPCs/radial glia, interneurons, oligodendrocytes, microglia, astrocytes, endothelium	Injury may arise from progenitor mistiming, migration scaffold defects, oligodendrocyte iron stress, microglial pruning, astroglial reactivity, or BBB disruption	scRNA-seq, lineage markers, complement, cytokines, TfR1/DHHC5, TEER	Cell-type-resolved assays separate direct neuronal toxicity from glial/vascular amplification	(Jiang et al., 2021; Liu et al., 2025; Yang et al., 2024; Xu et al., 2023)
Model architecture	2D cultures, organoids, chips, chimeric/animal models, clinical cohorts	Each model captures a different level: cell-autonomous injury, human developmental trajectory, immune-vascular biology, systemic physiology, or population risk	Morphology, omics, MEA, BBB/TEER, PK/PD, behavior, biomarkers	Integrate results by model validity domain instead of treating discrepancies as contradictions	(Zhao et al., 2025; Saglam-Metiner et al., 2024a; Johnson et al., 2019; Bleese et al., 2023)
Exposure delivery	Static bath, volatile gas-liquid exposure, vascular-like perfusion, chip materials, BBB barriers	Actual tissue dose depends on diffusion, gas stability, adsorption, washout, binding, and barrier permeability	Measured media/tissue concentration, flow, TEER, recovery sampling, adsorption controls	Report engineering details to distinguish biology from under/overexposure or device artifact	(Carstens et al., 2025; Saglam-Metiner et al., 2024a; Castiglione et al., 2022; Wang et al., 2024b; Saglam-Metiner et al., 2024b)
Patient susceptibility	Developmental age/maturity, biological sex, prematurity, congenital or genetic disease, baseline inflammation/metabolic state, genetic background, patient-derived organoids, isogenic controls, pediatric PK/PD	Developmental stage, neurotransmitter maturation, endocrine/neuroimmune state, metabolic reserve, baseline neural vulnerability, and drug exposure	Isogenic comparison, multi-omics, mitochondrial reserve, network resilience	Move from population-average risk toward susceptibility-stratified interpretation	(Sabitha et al., 2021; Wang et al., 2025; Zhao et al., 2020; Packiasabapathy et al., 2020; Anderson et al., 2020)

## 6 Susceptibility-informed preclinical risk modeling using organoid and chip platforms

### 6.1 Multidimensional response fingerprints for preclinical hypothesis generation

The preceding sections indicate that anesthetic developmental neurotoxicity cannot be adequately described by a single endpoint, such as apoptosis, reduced synaptic protein expression, or impaired behavioral performance. For organoid and chip-based studies, a more useful framework is to define multidimensional response fingerprints that map each exposure across drug class, dose or concentration, duration, developmental stage, brain-region identity, model architecture, and cellular composition. This approach is consistent with broader developmental neurotoxicity frameworks showing that different molecular initiating events may converge on common key events, such as altered BDNF signaling, impaired synaptogenesis, and disrupted neuronal network function, while still producing context-dependent phenotypes (Pistollato et al., 2020). In this setting, a response fingerprint should not be interpreted as a direct clinical risk score, but as a structured way to describe which biological layer is perturbed, under which exposure condition, and in which developmental model.

For anesthetic studies using human brain organoids, bMPSs, or organ-on-chip systems, such fingerprints can be constructed by combining structural, molecular, cellular, metabolic, and functional readouts. Structural features may include organoid size, ventricular zone-like organization, radial glial architecture, neuronal migration, regional identity, and lineage composition. Molecular and cellular features may include transcriptomic stress signatures, oxidative injury, mitochondrial respiration, calcium signaling, apoptosis, autophagy, ferroptosis, neuroinflammatory activation, and glial maturation. Functional readouts, including calcium imaging, multi-electrode arrays, and graph-based network analysis, can further quantify firing synchrony, burst structure, connectivity, excitatory–inhibitory balance, and activity-dependent plasticity. Recent developmental neurotoxicity discussions emphasize that new approach methodologies should move beyond isolated endpoints toward integrated test batteries covering proliferation, migration, differentiation, neurite outgrowth, synaptogenesis, myelination, and network formation/function (Celardo et al.). Similarly, human brain organoid-based risk assessment increasingly highlights the need to connect molecular and cellular substrates of plasticity with learning- and memory-relevant network phenotypes (Mohapatra et al., 2026).

A fingerprint-based approach is useful because developmental responses may be delayed, compensatory, or cell type-specific. For example, single-cell profiling of human neural organoids exposed to blood showed temporally dynamic and cell type-specific transcriptomic injury, with intermediate progenitors, endothelial cells, and astrocytes showing strong responses after longer exposure (Seah et al., 2025). Although this model is not anesthetic-specific, it illustrates that the dominant response may shift with exposure duration and cellular composition. MEA-based graph deviation analysis in human forebrain organoids also shows how network-level signatures can reveal early neurodevelopmental abnormalities not captured by morphology alone (Mencattini et al., 2025). Transcriptome-based profiling of cerebral organoids exposed to soman further supports the value of omics-defined toxicity signatures for identifying regulatory networks and candidate mechanisms (Wei et al., 2026).

Accordingly, future anesthetic organoid and chip studies should report exposure-aware profiles rather than binary toxic/non-toxic outcomes. A short sevoflurane exposure in an early cortical organoid, prolonged propofol exposure in a forebrain assembloid, and chronic midazolam exposure in an immune–vascular brain-on-chip model should be interpreted within different experimental contexts. Each profile should be positioned within a matrix of drug × dose or concentration × duration × developmental stage × brain region × model design × donor background. Such profiling can help distinguish transient adaptation from persistent lineage remodeling, acute cellular stress from delayed network dysfunction, and general exposure effects from susceptibility-associated responses. At this stage, multidimensional response fingerprints are best viewed as tools for mechanism classification, candidate biomarker discovery, and hypothesis generation before validation in animal models and clinical cohorts.

### 6.2 Developmental age, biological sex, and comorbidities as susceptibility modifiers

Developmental susceptibility to anesthetic neurotoxicity is unlikely to be determined by exposure characteristics alone. Age and maturational stage, biological sex, and pre-existing medical conditions can alter baseline neural development, pharmacokinetics and pharmacodynamics, inflammatory and metabolic state, and the capacity of the developing brain to compensate for pharmacological stress. These variables should therefore be treated as susceptibility modifiers rather than as independent determinants of toxicity. Importantly, current evidence does not support a simple rule in which a particular age, sex, or comorbidity uniformly predicts anesthetic-related neurodevelopmental injury. Instead, their effects interact with anesthetic class, cumulative exposure, developmental window, surgery, physiological instability, and underlying disease.

Developmental age is one of the strongest biological determinants of the type of response that can be produced by anesthetic exposure. As discussed above, early developmental windows are characterized by rapid changes in progenitor proliferation, neuronal migration, GABAergic and glutamatergic signaling, synaptogenesis, gliogenesis, and myelination, such that the cellular target of an exposure changes with maturation. Experimental evidence supports the existence of age-dependent vulnerability windows. In male rats, isoflurane exposure on postnatal day 7 produced later spatial and recognition-memory abnormalities, whereas delaying exposure until postnatal day 14 preserved spatial memory; androgen-receptor blockade or inhibition of the chloride importer NKCC1 also attenuated functional abnormalities (Chinn et al., 2020). These findings link age-dependent susceptibility to the developmental state of inhibitory neurotransmission rather than chronological age alone. In humans, however, a sharp age threshold for anesthetic neurotoxicity has not been established. The GAS trial found no broad neurodevelopmental impairment after a single approximately 1-h sevoflurane-based anesthetic exposure in infancy (McCann et al., 2019), whereas observational studies of younger, repeated, or prolonged exposure remain difficult to interpret because age at anesthesia is closely correlated with surgical indication, disease severity, and cumulative exposure.

Biological sex may further modify developmental responses. In neonatal rats exposed to sevoflurane, both sexes exhibited an acute increase in corticosterone, but long-term neuroendocrine and neurobehavioral abnormalities were more prominent in males; only males showed persistent increases in hippocampal CA1 inhibitory synaptic activity and abnormalities in anxiety-related behavior and prepulse inhibition (Xu et al., 2015). The age-dependent isoflurane study described above further implicated androgen signaling and NKCC1/KCC2-dependent chloride homeostasis in male susceptibility. More recent work also found weaker spatial-memory impairment after neonatal sevoflurane exposure in female rats, accompanied by sex-specific patterns of C3/TLR4 signaling, microglial polarization, and hippocampal synaptic changes (Cheng et al., 2025). Together, these studies suggest that sex steroids, developmental chloride gradients, neuroendocrine signaling, and neuroimmune responses may contribute to sex-dependent vulnerability. However, the direction and magnitude of sex effects vary among exposure paradigms, and biological sex should therefore be included as an experimental variable rather than interpreted as a fixed risk hierarchy.

Prematurity represents an especially important clinical susceptibility context because preterm infants combine developmental immaturity with a high burden of neurological, respiratory, inflammatory, and perioperative risk factors. A recent systematic review and meta-analysis found that preterm infants undergoing surgery under general anesthesia before 44 weeks postmenstrual age had a higher prevalence of MRI-detected brain abnormalities and lower neurodevelopmental scores than preterm infants without such exposure. However, all included studies were observational, and the evidence was rated as very low quality because surgery and anesthesia could not be separated from underlying illness and other uncontrolled confounders (Heisel et al., 2025). Independent of anesthesia, long-term studies of children born very preterm identify brain injury, bronchopulmonary dysplasia, biological sex, and socioeconomic context among important predictors of later neurodevelopmental outcomes (Burnett et al., 2026). Prematurity should therefore be viewed as a composite vulnerability state rather than as evidence that anesthetic agents are intrinsically more neurotoxic in preterm infants.

Congenital heart disease (CHD) provides another example in which anesthetic exposure occurs within a highly complex baseline risk environment. In infants undergoing congenital cardiac surgery, cumulative volatile-anesthetic exposure was not independently associated with lower neurodevelopmental scores at 18 months, whereas greater ketamine exposure was associated with poorer motor performance (Simpao et al., 2023). Importantly, neurodevelopmental outcome in CHD is itself associated with multiple medical and social factors, including genetic and cardiac diagnosis, birth weight, biological sex, age at surgery, hospital length of stay, number of catheterizations, and caregiver or neighborhood-level socioeconomic factors (Seed et al., 2026). These findings underscore that developmental abnormalities in medically complex children cannot be attributed to anesthetic neurotoxicity without careful adjustment for baseline disease and perioperative confounding.

These susceptibility variables also have implications for human organoid and bMPS design. Developmental age should be approximated using organoid maturation state and lineage composition rather than culture duration alone. Biological sex should be recorded for donor-derived lines, while acknowledging that isolated organoids do not reproduce the complete endocrine environment that contributes to *in vivo* sex differences. Comorbid states can be modeled using patient-derived or isogenic organoids and, where appropriate, by introducing defined secondary stressors such as inflammatory signaling, hypoxia, mitochondrial dysfunction, or altered metabolic conditions. Experimental designs should compare baseline phenotypes before anesthetic exposure and quantify whether the same exposure produces differential changes in progenitor dynamics, mitochondrial reserve, inflammatory signaling, synaptic maturation, and network function across susceptibility backgrounds. Such interaction-based designs are more informative than labeling individual patient characteristics as categorical predictors of anesthetic neurotoxicity.

### 6.3 Patient-derived organoids and susceptibility stratification

A limitation of population-level risk assessment is that it averages across individuals with different developmental trajectories, genetic backgrounds, and cellular stress thresholds. For anesthetic developmental neurotoxicity, vulnerability is unlikely to be determined by anesthetic exposure alone. It may instead arise from interactions among exposure parameters and pre-existing biological susceptibility, including rare pathogenic variants, common risk alleles, polygenic background, prematurity, metabolic state, inflammation, and neurodevelopmental disease liability. Patient-derived iPSC and organoid platforms provide a way to model these interactions in a human genetic context. Studies of rare neurodevelopmental disorders have shown that patient-derived iPSCs, CRISPR/Cas9 gene editing, and three-dimensional brain organoids can reveal abnormalities in progenitor behavior, neuronal differentiation, glial development, synaptic maturation, and molecular signaling that are difficult to access in patient brain tissue or conventional animal models (Sabitha et al., 2021).

Recent organoid biobank and atlas efforts further support the shift from generic models toward susceptibility-aware platforms. A large neurodevelopmental disorder iPSC resource combining genetically diverse patient-derived lines, clinical information, brain imaging, and genomic data showed that brain organoids can capture conserved and disease-category-specific phenotypes across microcephaly, polymicrogyria, epilepsy, and intellectual disability. Patient organoids displayed distinct cellular defects, including altered cell survival, intermediate progenitor abnormalities, excessive astrogliosis, and excessive TTR-positive cell generation, linking genotype, clinical phenotype, and organoid-level developmental readouts (Wang et al., 2025). For anesthetic studies, such baseline organoid phenotypes should not automatically be treated as experimental noise; they may represent susceptibility states that modify exposure responses.

Disease-specific organoid studies illustrate how this concept could be applied cautiously. In dup15q syndrome, single-cell analysis of patient-derived cortical organoids and postmortem brain tissue identified increased glycolysis, disrupted layer-specific marker expression, and abnormal deep-layer neuron morphology during fetal-stage development, together with postnatal synaptic-signaling burdens in upper-layer neurons (Perez et al., 2025). These findings are relevant because anesthetics can also perturb metabolism, synaptic signaling, and network excitability. Similarly, KMT2D-deficient and Kabuki syndrome patient-derived neural-crest-containing organoids revealed neural crest defects, GABAergic overproduction, and WNT3A enhancer dysregulation, while WNT signaling reactivation rescued lineage abnormalities (Shan et al., 2024). These examples suggest that patient organoids may help identify vulnerable cell types and modifiable developmental pathways, but they do not by themselves establish clinical anesthetic risk.

A susceptibility-informed testing pipeline could compare control, patient-derived, and isogenic-corrected organoids under matched exposure conditions. Isogenic controls are particularly important because they help separate variant-associated vulnerability from unrelated donor background. This principle is illustrated by APOE4 cerebral organoid models, in which APOE ε4/ε4 exacerbated apoptosis, synaptic loss, tau pathology, and stress granule formation, whereas isogenic conversion of APOE4 to APOE3 attenuated disease-related phenotypes (Zhao et al., 2020). Although this example concerns Alzheimer’s disease rather than anesthetic exposure, it provides a template for testing whether a risk genotype amplifies or reduces injury-related readouts under controlled conditions. Patient-derived models have also revealed variant-specific abnormalities in neuronal differentiation, calcium signaling, spontaneous activity, and neurotransmitter responsiveness, as shown in GNAO1 p.G203R iPSC-derived cortical neurons (Benedetti et al., 2024).

For anesthetic developmental neurotoxicity, patient-derived organoids are most appropriately positioned as susceptibility-modeling tools for identifying genetic or disease-associated differences in exposure response. Robust study design will require isogenic controls when feasible, multiple donors per group, replicate organoid batches, standardized exposure paradigms, blinded readout analysis, and comparison with animal or clinical biomarker data. In this framework, patient-derived and isogenic organoids can help define genetic or disease-associated contexts in which developmental stress responses are amplified, attenuated, or redirected. Their main value is to support preclinical hypothesis generation, candidate biomarker prioritization, and susceptibility-informed model benchmarking before *in vivo* and clinical validation.

### 6.4 Biomarkers and organoid-guided protective strategy screening

The value of mechanistic profiling lies in linking organoid-defined responses to measurable and temporally resolved markers of developmental neurotoxicity. Importantly, no single molecular, genetic, or biochemical marker is specific for anesthetic developmental neurotoxicity. Many candidate markers also change during normal neural maturation, adaptive stress responses, inflammation, or nonspecific cellular injury. Their interpretive value therefore depends on the direction and magnitude of change, cell type, developmental stage, exposure paradigm, and recovery interval. A panel-based approach that combines developmental, transcriptional, metabolic, cell-death, neuroimmune, synaptic, and functional readouts is more informative than reliance on an isolated marker (Zhao et al., 2020; Packiasabapathy et al., 2020; Anderson et al., 2020).

For practical interpretation, candidate markers can be organized into operational early, late, and delayed response windows. Here, “early” refers to changes detected during exposure or shortly after exposure, generally within hours to approximately 24 h; “late” refers to abnormalities that emerge or persist during the early recovery period over subsequent hours to days; and “delayed” refers to persistent developmental, epigenetic, structural, or network-level abnormalities detected after longer recovery intervals ranging from days to weeks or months, depending on the experimental model. These boundaries are not fixed biological thresholds and should not be transferred directly between organoids, rodents, non-human primates, and humans. Rather, they provide a framework for distinguishing initiating stress responses from persistent tissue remodeling and delayed functional consequences. Representative molecular, genetic, biochemical, and functional markers are summarized in Table 4.

**TABLE 4:** Time-resolved candidate markers for anesthetic developmental neurotoxicity assessment.

Temporal phase	Marker domain	Representative markers/readouts	Biological interpretation	Representative evidence
Early	Developmental/progenitor	SOX2, PAX6, Ki67/PH3, N-cadherin; Jag1/NICD; early shifts in TUJ1 and MAP2	Progenitor maintenance, proliferation, ventricular-zone integrity, Notch signaling, premature or altered neuronal differentiation	(Lee et al., 2022; Jiang et al., 2021; Dong et al., 2012; Song et al. 2022)
Early	Genetic/transcriptional	c-Fos, EGR1; exposure-stage-dependent neurodevelopmental and synaptic gene signatures	Immediate-early neuronal activity and adaptive transcription; early perturbation of developmental gene programs	(Wang et al., 2026; Kidambi et al., 2009)
Early	Mitochondrial/metabolic	ATP, mitochondrial membrane potential, intracellular Ca^2+^, CKMT1B, mitochondrial respiratory function	Acute bioenergetic failure, calcium dysregulation, and mitochondrial stress	(Pant et al., 2026; Sun et al., 2022; Zhang and Li, 2023; Liang et al., 2022; Ebrahimi et al., 2024)
Early	Oxidative/ferroptotic	ROS, GPX4, Nrf2, lipid peroxidation, iron accumulation	Oxidative stress, impaired antioxidant defense, and initiation of ferroptotic injury	(Song et al. 2022; Xia et al., 2018; Liu et al., 2025)
Early	Nitric oxide/cell death	nNOS, iNOS, PSD-95–NMDAR–nNOS signaling; BAX/BCL-2, cytochrome c, cleaved caspase-3, TUNEL; p-PERK, GRP78, ATF4, CHOP, caspase-12	Disturbance of physiological NO signaling and initiation of mitochondrial- or ER stress-associated apoptotic injury	(Li et al., 2026; Sun et al., 2022; Zhang and Li, 2023; Liang et al., 2022; Ebrahimi et al., 2024; Zhang et al., 2024; Feng et al., 2012; Schaefer et al., 2019; Zhao et al., 2026)
Early-to-late	Neuroimmune/inflammatory	IL-1β, IL-6, TNF-α, NF-κB, cGAS/STING, AIF1, CD11b, CD68	Microglial activation, inflammatory amplification, and neuron–glia injury signaling	(Saglam-Metiner et al., 2024a; Zhang et al., 2024; Yang et al., 2024; Liu et al., 2019; Xu et al., 2023)
Late	Lineage/glial maturation	TUJ1, MAP2, TH, TBR1, CTIP2, SATB2; GFAP, S100B; OLIG2, O4, MBP	Persistent shifts in neuronal subtype specification, astroglial maturation, oligodendrocyte development, and myelination	(Lee et al., 2022; Shang et al., 2022; Li et al., 2026; Saglam-Metiner et al., 2024a; Song et al. 2022)
Late	Synaptic/remodeling	DLG4/PSD-95, synaptic puncta or proteins, C1q/C3, microglial synaptic engulfment	Synaptic maturation or loss, complement-mediated pruning, and altered neuron–glia interactions	(Saglam-Metiner et al., 2024a; Schaefer et al., 2019; Xu et al., 2023)
Late-to-delayed	Non-apoptotic cell death	GPX4 and lipid peroxidation; NLRP3-related signaling; RIPK1/RIPK3/MLKL	Persistent or secondary ferroptotic, pyroptotic, or necroptotic injury	(Xia et al., 2018; Liu et al., 2025; Bai et al., 2025; Zhang et al., 2024)
Delayed	Genetic/epigenetic	Persistent chromatin-accessibility changes; mRNA/lncRNA signatures; CKMT1B-associated expression changes	Stable transcriptional or epigenetic reprogramming after the acute exposure period	(Pant et al., 2026; Doi et al., 2021)
Delayed	Structural/glial	MBP/myelination, oligodendrocyte maturation, dendritic complexity, synaptic density	Persistent remodeling of white matter, neuronal morphology, and synaptic architecture	(Li et al., 2026; Liu et al., 2025; Wang et al., 2024a; Xu et al., 2023)
Delayed	Functional/network	Calcium-network activity; MEA firing rate, burst structure and synchrony; oscillatory activity and network coupling	Persistent circuit maturation defects and functional network dysregulation	(Li et al., 2026; Carstens et al., 2025; Wang et al., 2024a)
Translational candidate	Circulating molecular	Exposure-associated circulating mRNA/lncRNA signatures overlapping with organoid responses	Potential bridge between human 3D phenotypes and clinically accessible biomarkers; requires independent validation	(Pant et al., 2026)

The temporal classification in Table 4 also illustrates why longitudinal sampling is essential. An early increase in ROS or cleaved caspase-3 may resolve without persistent developmental consequences, whereas transient progenitor or transcriptional perturbations may precede delayed changes in lineage composition, myelination, synaptic organization, or network synchrony. Conversely, delayed functional abnormalities can occur without prominent early apoptosis, as illustrated by nitric oxide-dependent synaptic plasticity defects and other sublethal developmental mechanisms (Schaefer et al., 2019). Thus, marker selection should pair at least one early molecular or biochemical readout with later developmental and functional endpoints whenever experimental design permits. Behavioral and cognitive abnormalities in animal or clinical studies should be regarded as delayed validation outcomes rather than molecular biomarkers.

Organoid and chip platforms may also help prioritize protective strategies before *in vivo* or clinical validation. Preclinical studies have examined mitochondrial stabilizers, antioxidants, anti-inflammatory compounds, trophic pathway modulators, epigenetic interventions, anesthesia-sparing strategies, and novel anesthetic classes (Useinovic et al., 2022). Clinically oriented reviews further discuss dexmedetomidine-based anesthetic sparing, opioid adjuncts to reduce volatile anesthetic dose, neuraxial or regional anesthesia when appropriate, and physiological optimization of cerebral oxygenation, hemodynamics, ventilation, temperature, glucose control, and electroencephalography-guided anesthetic depth (Andropoulos, 2023). Organoid and chip platforms can help prioritize candidates that reduce human cellular injury signatures without disrupting developmental programs for subsequent translational evaluation.

RNA-targeted strategies may be a future extension of this prioritization framework. miRNA-based approaches are attractive because miRNAs regulate apoptosis, oxidative stress, neuroinflammation, synaptic plasticity, and neurodevelopmental signaling; engineered agomirs or antagomirs may therefore modulate anesthetic-associated injury pathways (Minz et al., 2023). Antisense oligonucleotide approaches can also target RNA expression or splicing with high specificity, and patient-derived cerebral organoids may provide a human platform to evaluate efficacy, cell-type delivery, off-target effects, and toxicity before translation (Lange et al., 2022). These strategies remain far from routine perioperative use, but they illustrate how susceptibility models could support candidate intervention prioritization rather than immediate individualized treatment.

A practical translational pipeline would proceed in four steps: define the response fingerprint induced by a given exposure; identify molecular or functional biomarkers that track this fingerprint; test candidate protective strategies in control, patient-derived, and isogenic organoids; and validate robust signals in animal models and prospective clinical cohorts. Such a pipeline would support mechanism-guided and exposure-aware preclinical risk modeling, while avoiding the premature use of organoid results as direct clinical decision tools.

## 7 Current challenges and future perspectives

### 7.1 Quality control, reproducibility, and physiological maturation

A major challenge in applying human brain organoids to anesthetic developmental neurotoxicity assessment is the balance between developmental complexity and experimental reproducibility. Brain organoids can recapitulate human developmental programs, three-dimensional cytoarchitecture, progenitor dynamics, neuronal differentiation, and early network activity, but their self-organizing nature introduces variability in size, morphology, regional identity, cellular composition, maturation state, and baseline stress. For anesthetic studies, this variability is especially problematic because expected phenotypes, such as altered progenitor proliferation, premature differentiation, apoptosis, mitochondrial stress, synaptic disruption, or network changes, may be subtle and exposure-window dependent. Engineering strategies can improve reproducibility by controlling initial geometry, niche composition, matrix properties, microfluidic access, and functional readouts (Hofer and Lutolf, 2021). However, excessive standardization may reduce intrinsic self-organization, leaving an unresolved tension between developmental fidelity and assay robustness.

Recent platforms provide partial solutions. Single-rosette cortical organoids reduce multi-rosette heterogeneity and generate reproducible early neuroepithelial structures suitable for detecting teratogen-sensitive developmental phenotypes (Tidball et al., 2023). Hi-Q brain organoids improve scalability by producing large numbers of organoids with consistent size, cytoarchitecture, cell diversity, functionality, low ectopic stress-pathway activation, and compatibility with drug screening (Ramani et al., 2024). Long-term advanced cerebral organoid protocols and air–liquid interface cultures improve nutrient access, neuronal survival, axon outgrowth, and electrophysiological maturation, although batch effects and line-specific biases remain (Giandomenico et al., 2021). For neurodevelopmental disease modeling, organoids retain patient-relevant genetic backgrounds, but organoid-to-organoid variability, limited representation of some resident brain cell types, and long maturation times still restrict interpretation (Wang et al., 2023).

Future anesthetic studies should therefore define a minimum quality-control framework before exposure. Essential parameters include donor or cell line information, organoid protocol, culture day, biological maturation stage, organoid size, regional identity, cell composition, baseline stress level, and spontaneous activity. Key QC markers should cover radial glia, intermediate progenitors, neurons, astroglia, synapses, and functional network maturation. Automated imaging, multi-omics, machine learning, and quantitative high-throughput 3D screening may further improve cross-laboratory comparability (Wang and Jeon, 2022). In this way, organoid platforms can move from descriptive developmental models toward standardized, benchmarkable systems for exposure-aware anesthetic neurotoxicity testing.

### 7.2 Benchmarking neurovascular, immune, and circuit-level complexity

A second unresolved gap is that most brain organoid models remain neuron-centered, whereas anesthetic exposure *in vivo* occurs within a neurovascular, neuroimmune, and circuit-forming developmental environment. Missing vasculature can create diffusion limits, hypoxia, necrosis, and metabolic stress, which may confound endpoints used to infer anesthetic toxicity. ETV2-induced vascularized human cortical organoids form vascular-like networks, show improved maturation, and acquire partial blood–brain barrier-related features, including tight-junction and transporter expression (Cakir et al., 2019). Vascularized cortical organoids can also support prolonged culture, synaptic activity, and improved graft survival after transplantation (Shi et al., 2020). However, current vascularized human brain organoids only partially model neurovascular development and early barriergenesis; most still lack sustained blood-like flow and a mature functional blood–brain barrier (Kistemaker et al., 2025). Therefore, they are better suited for testing neurovascular susceptibility mechanisms than for claiming complete pharmacokinetic or BBB modeling.

Transplantation provides a complementary validation layer. Human brain organoids grafted into mouse brain become vascularized, mature further, integrate host microglia, extend axons, show functional neuronal networks, and display graft-to-host connectivity (Mansour et al., 2018). This improves physiological support but also introduces a cross-species environment that complicates human-specific interpretation. Microglia represent another key missing component. Human iPSC-derived microglia-like cells can secrete cytokines, migrate, phagocytose CNS substrates, and integrate into organoids (Abud et al., 2017). Microglia-sufficient organoids show that iPSC-derived primitive macrophages can adopt microglia-like phenotypes and promote neuronal maturation through cholesterol transfer (Park et al., 2023). *In vivo* neuroimmune organoids further demonstrate that human microglia can acquire human-specific signatures, survey the organoid environment, and respond to injury and systemic inflammatory cues (Schafer et al., 2023). For anesthetic studies, these models are important because toxicity may involve glial metabolism, inflammatory priming, synaptic pruning, and neuron–glia lipid signaling, not only direct neuronal damage.

Circuit-level complexity also requires careful platform selection. Assembloids and region-specific fusion models can reconstruct selected inter-regional interactions, interneuron migration, excitatory–inhibitory balance, myelination, and long-range activity (Maisumu et al., 2025). The specific advantages and limitations of assembloids compared with conventional brain organoids for preclinical neurotoxicity studies are discussed below and summarized in Table 5.

**TABLE 5:** Advantages and limitations of human brain organoids and assembloids for preclinical neurotoxicity studies.

Platform	Major advantages	Major limitations	Most appropriate neurotoxicity applications	Refs
Cerebral and region-specific brain organoids	Human genetic and developmental context; 3D progenitor and lineage organization; region-specific differentiation; multimodal molecular, cellular, metabolic, and electrophysiological readouts; patient-derived and isogenic modeling; controlled dose, duration, and recovery paradigms; increasing compatibility with screening	Organoid-to-organoid and batch variability; incomplete and heterogeneous maturation; limited vascular, immune, and late glial representation; diffusion-related hypoxia or metabolic stress; long culture duration; no systemic PK/PD, maternal–fetal physiology, metabolism, behavior, or whole-brain connectivity	Developmental trajectory, lineage-specific toxicity, mitochondrial/redox injury, programmed cell death, synaptic maturation, regional susceptibility, biomarkers, mechanistic rescue and neuroprotective screening	(Birtele et al., 2025; Camp et al., 2015; Velasco et al., 2019; Wang et al., 2023; Quadrato et al., 2017; Trujillo et al., 2019; Gordon et al., 2021; Hofer and Lutolf, 2021; Tidball et al., 2023; Ramani et al., 2024; Giandomenico et al., 2021; Wang and Jeon, 2022; Kistemaker et al., 2025; Hogberg and Smirnova, 2022; Liu et al., 2026)
Assembloids/fused region-specific organoids	Reconstruct selected inter-regional interactions; model interneuron migration and functional integration; assess excitatory–inhibitory balance, axonal connectivity, myelination, and network synchronization; enable circuit-level neurotoxicity questions not accessible in isolated organoids	Variability from each component plus fusion timing, size, orientation, and maturation mismatch; greater technical complexity and cost; lower throughput; more difficult exposure standardization and causal attribution; diffusion limitations; still lack complete vascular, immune, endocrine, and systemic physiology	Migration defects, cross-region susceptibility, disrupted E/I balance, altered circuit formation, inter-regional connectivity and network-level developmental toxicity	(Birey et al., 2017; Maisumu et al., 2025)

### 7.3 Advantages and limitations of organoids and assembloids for preclinical neurotoxicity studies

Brain organoids occupy an intermediate position between reductionist two-dimensional neural cultures and whole-animal models. Their principal advantage for developmental neurotoxicity research is the ability to study human-specific developmental processes within a three-dimensional tissue context while retaining experimental control over exposure concentration, duration, developmental stage, and recovery interval. Cerebral and region-specific organoids reproduce selected fetal and early postnatal developmental programs, including progenitor-zone organization, neuronal and glial differentiation, synaptic maturation, and emerging electrophysiological activity (Birtele et al., 2025; Camp et al., 2015; Velasco et al., 2019; Wang et al., 2023; Quadrato et al., 2017; Trujillo et al., 2019; Gordon et al., 2021). They can also be generated from patient-derived or genetically engineered pluripotent stem cells, enabling comparison of donor-specific or isogenic susceptibility backgrounds. Combined with transcriptomics, single-cell analysis, mitochondrial assays, calcium imaging, and MEA recording, organoids allow neurotoxicity to be characterized across molecular, cellular, developmental, and functional levels rather than by cytotoxicity alone. Their compatibility with repeated sampling, mechanistic perturbation, and emerging scalable culture formats further supports their use for dose–response analysis, biomarker discovery, and candidate neuroprotective screening (Hofer and Lutolf, 2021; Tidball et al., 2023; Ramani et al., 2024; Giandomenico et al., 2021; Wang and Jeon, 2022).

These advantages are accompanied by important limitations. Organoid self-organization can introduce variability in size, regional identity, cell composition, developmental maturity, and baseline stress, although guided and engineered protocols can substantially improve reproducibility (Velasco et al., 2019; Hofer and Lutolf, 2021; Tidball et al., 2023; Ramani et al., 2024; Giandomenico et al., 2021; Wang and Jeon, 2022). Standard brain organoids generally remain developmentally immature and incompletely reproduce vascular, immune, mesoderm-derived, and late-maturing glial components. Limited diffusion can generate hypoxia, metabolic stress, or necrotic regions that may confound interpretation of neurotoxic endpoints. Moreover, static organoids do not reproduce systemic pharmacokinetics, placental or hepatic metabolism, endocrine regulation, physiological compensation, or behavioral outcomes. These limitations are particularly relevant to anesthetic studies because volatile and lipophilic agents may show uncertain tissue partitioning, and nominal culture-medium concentrations may not reflect actual tissue exposure. Therefore, organoids are most informative for studying human developmental mechanisms and cellular susceptibility rather than for directly predicting whole-organism or clinical neurotoxicity (Birtele et al., 2025; Kistemaker et al., 2025; Hogberg and Smirnova, 2022; Liu et al., 2026).

Assembloids extend organoid models by combining separately patterned neural regions or cell populations to reconstruct selected inter-regional interactions. Fusion of dorsal and ventral forebrain spheroids, for example, permits interneuron migration and functional integration into excitatory cortical circuits (Birey et al., 2017). More broadly, assembloid approaches can model excitatory–inhibitory interactions, neuronal migration, myelination, axonal connectivity, and network synchronization that cannot be adequately studied in isolated region-specific organoids (Maisumu et al., 2025). These features make assembloids particularly attractive when a neurotoxicant is hypothesized to disrupt circuit formation or communication between anatomically distinct developmental domains.

However, the additional biological complexity of assembloids also reduces experimental simplicity. Variability can arise not only within each constituent organoid but also from fusion timing, relative size, spatial orientation, cellular composition, and maturation mismatch between the assembled regions. Larger and more complex tissues may intensify diffusion limitations, while preparation time, cost, technical expertise, and quality-control requirements reduce throughput compared with simpler organoid or bMPS formats. The presence of multiple interacting compartments may also complicate causal attribution when an exposure-induced phenotype could originate in either region or from altered inter-regional communication. Most assembloids still lack complete vascular, immune, endocrine, and systemic physiological inputs and therefore remain modular circuit models rather than reconstructions of the whole human brain (Birtele et al., 2025; Maisumu et al., 2025). Accordingly, platform complexity should be matched to the biological question: simpler organoids are preferable for developmental trajectory and cell-autonomous mechanisms, whereas assembloids are most useful when migration, regional interaction, excitatory–inhibitory balance, or circuit-level functional connectivity is the primary endpoint.

### 7.4 Translational qualification, regulatory boundaries, and ethical governance

The third challenge is translating organoid and chip phenotypes into clinically and regulatorily meaningful evidence. Organoids can reveal developmental mechanisms, but altered cell markers, transcriptomic signatures, or network activity do not directly predict neurodevelopmental outcomes. Organoids-on-chips may improve translational relevance by combining organoid biology with microfluidic control of flow, oxygen, nutrient gradients, tissue interfaces, and real-time sensing (Wang H. et al., 2024). This is relevant to anesthetic research because clinical exposure is dynamic, involving changing drug concentrations, washout, possible metabolite accumulation, and fluctuations in oxygenation or inflammation. Patient-derived organoids-on-chips may support disease modeling and drug screening (Man et al., 2024), but they also introduce technical uncertainties, including device variability, polymer adsorption of lipophilic drugs, sensor calibration, limited throughput, and difficulty modeling volatile anesthetic partitioning. Therefore, future studies should report measured exposure in culture medium or tissue whenever possible, rather than relying only on nominal concentration.

Regulatory translation should position organoids and organ-on-chip systems within new approach methodologies and next-generation risk assessment, not as direct replacements for animal or clinical studies. New approach methodologies include organoids, microphysiological systems, omics, high-throughput assays, machine learning, and artificial intelligence, but regulatory adoption requires evidence of predictivity, reproducibility, quantification, and relevance to repeated-dose or chronic toxicity (Schmeisser et al., 2023). For anesthetic developmental neurotoxicity, useful pipelines should include adverse outcome pathway logic, reference anesthetics, positive and negative controls, multi-donor replication, blinded analysis, transcriptomic benchmark-dose modeling, and quantitative in vitro-to-in vivo extrapolation. Recent regulatory discussions also emphasize that reliability, applicability, and standard-setting remain essential for organoid and organ-on-chip use in drug development (Zhou et al., 2025).

Precision medicine is a future direction, but it should be framed cautiously. Patient-derived organoids may help model susceptibility contexts, such as mitochondrial fragility, neurodevelopmental risk variants, prematurity-related inflammation, or impaired stress resilience (Tao et al., 2025). However, they should be interpreted as susceptibility models, not deterministic predictors of clinical outcome. Ethical governance is also required because biobanks, pediatric or prenatal samples, multi-omics data, and susceptibility models raise issues of consent, privacy, and equitable access. More complex brain organoids also raise anticipatory ethical questions about advanced neural activity and possible “islands of awareness” (Bayne et al., 2020). Current organoids should not be assumed to be conscious, but vascularized, microglia-containing, stimulated, or transplanted models require transparent monitoring and oversight. Progress in this field should therefore combine mechanistic precision with caution in clinical messaging, regulatory claims, and ethical interpretation.

### 7.5 Practical clinical implications and future predictive risk modeling

Current evidence does not support a universal ranking of routinely used general anesthetics according to developmental neurotoxicity, and no single agent can presently be designated as the safest choice for the developing human brain. Anesthetic selection should therefore remain primarily guided by procedural requirements, established perioperative safety, age-appropriate pharmacokinetics and pharmacodynamics, patient comorbidities, and the anticipated exposure burden rather than by comparative neurotoxicity signals derived from individual animal or organoid studies. The GAS trial provides reassuring evidence that a single, relatively brief sevoflurane-based general anesthetic in infancy was not associated with broad neurodevelopmental impairment at 5 years (McCann et al., 2019). However, greater uncertainty remains for repeated or prolonged exposure and for biologically vulnerable populations. When clinically feasible, rational management should therefore focus on avoiding unnecessary repeated exposure and excessive duration while maintaining adequate hypnosis, analgesia, immobility, and physiological stability.

Practical risk reduction is therefore better framed as exposure- and physiology-aware anesthetic management than as avoidance of a specific anesthetic class. Techniques that reduce unnecessary anesthetic exposure may be considered when appropriate for the procedure and patient, but anesthetic-sparing should not itself be equated with developmental neuroprotection. This distinction is illustrated by a recent randomized clinical trial in children younger than 2 years. Addition of dexmedetomidine and remifentanil reduced mean end-tidal sevoflurane concentration from 2.6 to 1.8 vol%, yet no significant difference in intelligence, behavioral, or language-related outcomes was detected at 28–30 months; the prespecified primary endpoint of full-scale IQ at 5 years remains under follow-up (Ji et al., 2025). Therefore, reduction in exposure to one anesthetic agent should not be interpreted as established neuroprotection.

Pharmacological mitigation should be interpreted with similar caution. As discussed above, dexmedetomidine, melatonin, lithium, erythropoietin, coenzyme Q10, and other candidate interventions can attenuate selected molecular, cellular, or behavioral abnormalities in preclinical models (Sanders et al., 2009; Johnson et al., 2019; Bleese et al., 2023; Zhang et al., 2021; Yu et al., 2025; Zou et al., 2023; Noguchi et al., 2016; Goyagi, 2019), but no pharmacological agent is currently established for routine prophylaxis against anesthetic developmental neurotoxicity in children (Johnson et al., 2019; Bleese et al., 2023). Candidate protective strategies should therefore be evaluated through paired injury-and-rescue designs and should demonstrate recovery across multiple developmental domains rather than suppression of a single marker. Their own effects on neuronal differentiation, synaptic activity, hemodynamics, and development also require evaluation before clinical translation.

Future individualized prediction should distinguish baseline biological susceptibility from anesthetic exposure and perioperative physiological stress. Candidate preoperative variables include chronological and postmenstrual age, gestational age at birth and prematurity, biological sex, birth weight, baseline neurological or neurodevelopmental status, congenital cardiac or neurological disease, known genetic or mitochondrial disease, hepatic and renal function, active infection or systemic inflammation, concomitant neuroactive medications, and the number and cumulative duration of previous anesthetic or sedative exposures (Chinn et al., 2020; Xu et al., 2015; Cheng et al., 2025; Heisel et al., 2025; Burnett et al., 2026; Simpao et al., 2023; Seed et al., 2026; Park and Jang, 2026). Exposure-related variables may include anesthetic class, end-tidal or infusion concentration, cumulative dose or concentration–time exposure, procedure duration, repeated exposure, and concomitant sedative or analgesic agents (Simpao et al., 2023; Ji et al., 2025; Park and Jang, 2026). Intraoperative variables may include blood pressure and hypotension burden, oxygen saturation and hypoxemia burden, end-tidal carbon dioxide, cerebral oxygenation when available, electroencephalographic features, temperature, glucose, hemoglobin, and major perioperative complications (Park and Jang, 2026; Baek et al., 2026). Postoperative intensive-care exposure, prolonged sedation, candidate circulating biomarkers, and serial neurodevelopmental assessments could subsequently support dynamic risk updating (Pant et al., 2026; Simpao et al., 2023; Park and Jang, 2026). Importantly, these variables should initially be regarded as candidate predictors rather than established causal determinants of anesthetic developmental neurotoxicity. Social and environmental variables, including caregiver education and socioeconomic context, may improve prediction of long-term neurodevelopmental outcomes and should be considered as contextual predictors or confounders where appropriate, but they should not be interpreted as biological markers of anesthetic neurotoxicity (Burnett et al., 2026; Seed et al., 2026).

A transparent statistical starting point could model the probability of an adverse neurodevelopmental outcome as a function of baseline susceptibility, anesthetic exposure, perioperative physiological stress, and clinical context. Conceptually, this may be expressed as: $logit\left[P\left(Y=1\right)\right]=\beta 0+\beta SXS+\beta EXE+\beta \Phi X\Phi +\beta CXC+\beta SE\left(XS\times XE\right)$ , where Y represents the adverse developmental outcome, XS baseline patient susceptibility, XE anesthetic exposure burden, XΦ perioperative physiological stress, and XC clinical context. The interaction term XS × XE tests whether the association between anesthetic exposure and outcome differs according to baseline susceptibility. This equation is intended as a conceptual research framework rather than a validated bedside risk score. Potential statistical approaches include penalized regression methods such as ridge, least absolute shrinkage and selection operator, or elastic-net regression for high-dimensional correlated predictors. Mixed-effects or hierarchical Bayesian models may also be considered when repeated assessments, repeated exposures, or center-level heterogeneity are present. Prediction-model studies using either regression or machine-learning approaches should clearly define the predictors, outcome and prediction horizon, model-building procedures, internal validation, and performance measures such as discrimination and calibration, and should be transparently reported according to contemporary guidance such as TRIPOD + AI (TRIPOD AI, 2024).

Machine-learning approaches may extend these models when sufficiently large and representative datasets become available. Tree-based models such as random forests and gradient boosting can capture nonlinear interactions among patient characteristics, exposure variables, and physiological measurements, whereas temporal models such as long short-term memory networks and Transformers may be useful for high-frequency intraoperative time-series data. The feasibility of this approach has already been demonstrated for acute pediatric anesthetic risk prediction. Baek et al. used high-frequency vital-sign and ventilator data from two hospitals to develop and externally evaluate XGBoost, long short-term memory, InceptionTime, and Transformer models for prediction of intraoperative hypoxemia in children under general anesthesia (Baek et al., 2026). However, this model predicted an acute physiological event rather than developmental neurotoxicity, and its modest precision-related performance illustrates the difficulty of translating high-performing discrimination metrics into clinically reliable alerts.

More broadly, current AI applications in pediatric neuroanesthesia remain at an early stage and focus primarily on perioperative risk stratification, airway management, anesthetic-depth assessment, and real-time prediction of adverse physiological events rather than developmental neurotoxicity (Park and Jang, 2026). Future models will require multimodal integration of electronic medical records, physiological signals, neurophysiological monitoring, laboratory data, exposure history, and potentially validated molecular biomarkers. AI should be positioned as an explainable, physiology-informed decision-support tool that complements rather than replaces anesthesiologists’ clinical judgment.

Any future developmental neurotoxicity prediction program should therefore be developed as a clinical decision-support system rather than an automated anesthetic-selection algorithm. Development studies should prespecify clinically meaningful outcomes, clearly define predictors and prediction horizons, appropriately address missing data and class imbalance, and evaluate both discrimination and calibration. Models intended for broader clinical use should undergo rigorous evaluation in temporally and geographically independent cohorts, together with assessment of clinical utility, subgroup performance, fairness, interpretability, and generalizability. Development and evaluation studies should be transparently reported according to contemporary prediction-model guidance such as TRIPOD + AI (TRIPOD AI, 2024). Organoid-derived response fingerprints, omics signatures, or circulating biomarkers could eventually be incorporated as biological predictors, but only after reproducible associations with clinically meaningful outcomes have been established. At present, neither an organoid-derived score nor a machine-learning output should determine anesthetic choice for an individual child.

## 8 Discussion and conclusion

The evidence reviewed in this article supports a reframing of anesthetic developmental neurotoxicity. Rather than a single linear cascade from anesthetic exposure to neuronal death and later cognitive impairment, it is better understood as a context-dependent perturbation of human neurodevelopmental trajectories. Developmental disruption may occur through altered progenitor timing, premature or delayed differentiation, impaired neuronal migration, glial maturation defects, maladaptive synaptic pruning, abnormal network synchronization, and persistent metabolic or inflammatory remodeling. Broader developmental neurotoxicity literature similarly indicates that endocrine, immune, placental, gut–brain, and neuroplasticity pathways can influence developmental outcomes without producing classical neuropathology (Rock and Patisaul, 2018; Smirnova et al., 2014). For anesthetic research, the key question is therefore not whether a drug is globally neurotoxic, but under which exposure conditions, developmental windows, cellular contexts, and biological backgrounds it becomes disruptive. This framework positions human 3D neural models according to the developmental and mechanistic questions that each platform can reliably address. Integrating model capability with exposure variables, multidimensional readouts, and susceptibility factors provides a structured basis for comparing heterogeneous preclinical findings and prioritizing phenotypes for further translational validation.

Human brain organoids, brain microphysiological systems, and organ-on-chip platforms make this trajectory-based framework experimentally accessible by providing a human developmental substrate for controlled analysis of progenitor, neuronal, glial, metabolic, synaptic, and early network responses. BBB-integrated, immune-integrated, and sensor-compatible systems further extend this capability by introducing exposure control, tissue interfaces, and longitudinal functional readouts.

A central theme is the coexistence of convergence and divergence. Across anesthetic classes, current evidence points to shared platform-detectable stress modules, including progenitor disruption, mitochondrial dysfunction, oxidative injury, programmed cell death, ferroptosis, synaptic dysregulation, neuroimmune remodeling, and abnormal network activity. These modules interact rather than operate in isolation: mitochondrial injury can amplify oxidative stress and cell death; neuroinflammation can accelerate synaptic elimination; and altered progenitor dynamics can reshape later cellular composition. This interdependence supports the use of adverse outcome pathway thinking, toxicogenomic signatures, and key-characteristic frameworks to organize anesthetic developmental neurotoxicity as a network of interacting events rather than a single endpoint (Pistollato et al., 2020; Lein et al., 2026).

Divergent responses should not be dismissed as experimental noise. Sevoflurane, propofol, ketamine/HNK, and midazolam differ in receptor pharmacology, exposure route, dose or concentration stability, recovery interval, and clinical context. Their effects also depend on whether the modeled window corresponds to progenitor expansion, neuronal migration, synaptogenesis, myelination, or circuit refinement. A short exposure in an early cortical organoid, prolonged sedation in a BBB–immune–organoid chip, and repeated neonatal exposure in an animal model should not be expected to produce identical readouts. The field should therefore avoid forcing heterogeneous findings into a binary safe-versus-toxic conclusion. A more useful strategy is exposure-aware comparative profiling across drug, dose or concentration, duration, developmental stage, brain-region identity, lineage composition, model architecture, and donor background.

Clinical findings should therefore be interpreted within the exposure context outlined above: reassuring outcomes after a single short anesthetic exposure should not be generalized to repeated, prolonged, or biologically vulnerable settings (McCann et al., 2019; Xin et al., 2025). Conversely, preclinical injury signals should be used to generate and prioritize clinically testable hypotheses rather than interpreted as direct evidence of human neurodevelopmental harm. Organoid-based research can contribute by identifying developmental stress modules, susceptibility states, and candidate biomarkers that are difficult to detect in population-level clinical analyses. Pediatric anesthesia already recognizes that age, maturation, disease state, and drug interactions shape anesthetic response (Packiasabapathy et al., 2020; Anderson et al., 2020). Organoid and chip platforms extend this principle to human developmental neurotoxicity modeling.

Patient-derived and isogenic organoid systems are an important future direction, but their role should be framed cautiously. Genetic variants, mitochondrial fragility, prematurity, inflammation, neurodevelopmental disorders, and altered stress-resilience pathways may modify the response to anesthetic exposure. Patient-derived organoids can help model these contexts when combined with single-cell profiling, electrophysiology, mitochondrial assays, and biomarker discovery. However, they should be used as susceptibility models, not deterministic clinical decision tools. Their near-term value lies in hypothesis generation, mechanism prioritization, exposure comparison, candidate biomarker discovery, and protective strategy screening before validation in animal models and clinical cohorts.

Several methodological requirements must be addressed before these platforms can support translational qualification. Exposure design should report measured drug concentration when possible, especially for volatile or lipophilic anesthetics, together with exposure duration, stability, washout, recovery interval, and modeled developmental stage. Organoid quality control should include donor background, passage number, protocol, regional identity, cellular composition, maturation markers, batch variability, baseline stress, and functional activity. Platform complexity should be selected according to the question: simple organoids for cell-autonomous and developmental-trajectory endpoints, bMPSs for standardized 3D neural responses, BBB- or immune-integrated chips for exposure-interface biology, assembloids for circuit-level questions, and animal models for systemic and behavioral validation. Brain organoid-on-chip systems, 3D brain cultures, sensor-integrated platforms, and high-density MEA systems provide promising routes toward this goal (Carstens et al., 2025; Castiglione et al., 2022; Saglam-Metiner et al., 2024b; Hogberg and Smirnova, 2022). Ethical and regulatory interpretation must also remain cautious. More complex organoids raise governance questions regarding neural activity, while regulatory use will require reproducibility, predictivity, benchmark chemicals, and weight-of-evidence integration with animal and clinical data (Sachana et al., 2021; Castiglione et al., 2025).

In conclusion, anesthetic developmental neurotoxicity should be conceptualized as an exposure-aware and context-dependent disturbance of human neurodevelopment rather than a uniform drug effect. Human brain organoids and engineered neural platforms do not replace clinical studies or animal models, but they provide a mechanistic bridge between molecular injury pathways, human developmental biology, and translational hypothesis generation. Their greatest contribution will be to clarify why anesthetic responses converge on shared developmental stress modules yet diverge across drugs, stages, regions, models, and biological backgrounds. By combining patient-derived or isogenic organoids, microphysiological systems, multi-omics, electrophysiology, biomarker anchoring, and standardized exposure modeling, the field can move beyond generalized safety debates toward susceptibility-informed preclinical risk modeling and rational prioritization of neuroprotective strategies before *in vivo* and clinical validation (Chen et al., 2026; Liu et al., 2026; Alam El Din et al., 2024).
